# Accuracy of Medicare Information Provided by State Health Insurance Assistance Programs

**DOI:** 10.1001/jamanetworkopen.2025.2834

**Published:** 2025-04-01

**Authors:** Kacey Dugan, Ilse Peterson, Allison Dorneo, Melissa M. Garrido

**Affiliations:** 1 Faegre Drinker Biddle & Reath, Washington, DC; 2 Department of Health Law, Policy & Management, Boston University School of Public Health, Boston, Massachusetts; 3 Partnered Evidence-Based Policy Resource Center, Boston VA Healthcare System, Boston, Massachusetts; 4 Department of Surgery, Brigham and Women’s Hospital, Boston, Massachusetts

## Abstract

**Importance:**

Medicare beneficiaries, particularly those dually eligible for Medicaid, must navigate complex coverage options. The State Health Insurance Assistance Program (SHIP) provides counseling on Medicare options, but little is known about the quality of counseling sessions.

**Objective:**

To characterize experiences connecting to SHIP counselors, and to characterize the accuracy and completeness of information provided by counselors in response to questions about Medicare coverage options.

**Design, Setting, and Participants:**

In this cross-sectional study of 131 SHIP sites across the US, mystery shoppers posed as individuals newly eligible for Medicare. Shops occurred via telephone, in-person, and videoconference encounters from September 2023 to August 2024. Statistical analysis was performed from August to September 2024.

**Exposure:**

Mystery shoppers followed scripts with questions about coverage decisions corresponding to general Medicare eligibility and dual eligibility for Medicare and Medicaid.

**Main Outcomes and Measures:**

Responses were categorized as (1) accurate and complete, (2) accurate but incomplete, (3) not substantive, and (4) incorrect. This was a descriptive study without hypotheses.

**Results:**

Shoppers attempted 306 encounters. Of these, 122 (39.9%) could not be completed, most often because shoppers did not receive return calls. Within the 184 completed shops, the mean (SD) percentage of accurate and complete answers was 40.0% (25.7%). The percentage of responses with accurate answers (whether complete or incomplete) ranged from 26.1% (when asked whether a specific clinician was in network for a specific plan) to 94.3% (when asked about differences between traditional Medicare [TM] and Medicare Advantage [MA]). Responses were unlikely to be inaccurate (mean [SD], 6.7% [5.4%]). Fewer than half of counselors (44.8% [43 of 96]) mentioned Dual-Eligible Special Needs Plans (D-SNPs) as an option for mystery shoppers posing as dual eligibles.

**Conclusions and Relevance:**

In this study of the accuracy of Medicare information provided by SHIP counselors, shoppers encountered challenges in reaching SHIP sites, indicating possible capacity constraints; responses varied in accuracy and completeness, with better performance on questions about TM vs MA comparisons, and weaker performance on questions about integrated care plans and specific MA plan details. Given recent growth in MA and federal efforts to counter deceptive marketing practices from agents or brokers—in part by directing beneficiaries to SHIPs—policymakers should consider providing SHIP with additional resources for training and capacity improvements.

## Key Points

**Question:**

Do State Health Insurance Assistance Program (SHIP) counselors provide accurate and complete information to Medicare beneficiaries about their coverage options?

**Findings:**

In this cross-sectional study of 306 attempted mystery shopping encounters at 131 SHIP sites in 16 states, 39.9% could not be completed, often because shoppers did not receive return calls. In 184 completed encounters, the percentage of accurate answers ranged from 26.1% (when asked whether a specific clinician was in network) to 94.3% (when asked about differences between traditional Medicare and Medicare Advantage).

**Meaning:**

These results suggest that SHIPs may require additional resources to expand capacity and provide counselors with additional training to address the needs of Medicare beneficiaries seeking unbiased information on coverage options.

## Teaser

This cross-sectional study describes experiences of mystery shoppers connecting with State Health Insurance Assistance Program counselors via telephone, in-person, and videoconference encounters, and it evaluates the accuracy and completeness of information provided by counselors in response to questions about Medicare coverage options.

## Introduction

Medicare beneficiaries face an increasingly large and complex array of coverage options.^1^ Coverage option choice affects out-of-pocket costs and access to clinicians, medications, and services.^2^ However, complex insurance concepts and plan options and health literacy challenges can lead individuals to make suboptimal choices that limit medication access or incur higher costs.^3,4,5^

These challenges are particularly acute for individuals who are dually eligible for Medicare and Medicaid (dual eligibles). Dual eligibles are more likely than Medicare-only beneficiaries to be in poor health and have disabilities.^6^ They may be eligible for Dual-Eligible Special Needs Plans (D-SNPs)^7^ or other integrated options. Other low-income Medicare beneficiaries may be eligible for assistance through Medicare Savings Programs (MSPs) or the Part D Low-Income Subsidy (LIS).^8,9^

Resources available to assist Medicare beneficiaries in selecting coverage, including Medicare.gov and the 1-800-MEDICARE hotline, are difficult or undesirable for beneficiaries to use.^10^ Insurance brokers can provide helpful information but may have financial incentives to steer enrollees to suboptimal choices.^11^

The State Health Insurance Assistance Program (SHIP) has been highlighted by the federal government as a community-based, unbiased alternative to these resources.^12^ SHIP is administered by the Administration for Community Living (ACL) and receives approximately $70 million annually in federal funding (primarily from annual discretionary appropriations).^13^ Operating in 50 states, the District of Columbia, Guam, Puerto Rico, and the US Virgin Islands, SHIP provides grants to states. States subcontract with community-level organizations to maintain networks of trained certified paid and volunteer SHIP counselors responsible for providing free, one-on-one counseling to Medicare-eligible individuals.^3^

However, evidence indicates access gaps in SHIP services, including that SHIP sites are unevenly distributed, potentially limiting access to in-person counseling. All SHIP counselors must be certified by their state program and receive training on Medicare benefits, eligibility and coverage, and appeal procedures. A centralized curriculum is available from the National SHIP Technical Assistance Center,^14^ but each state has the flexibility to design their own training program. Interviews with SHIP staff suggest that some counselors may desire additional training on complex topics, including Medicaid coverage for dually eligible individuals.^15^ However, the quality of counseling sessions where SHIP services are available has not, to our knowledge, been systematically studied. Such evidence is needed to better understand how effective SHIP sites are in connecting Medicare beneficiaries to appropriate coverage and to identify opportunities for service improvement.

To obtain this evidence, we conducted a mystery shopper study of SHIP sites across the US. We sought to characterize experiences connecting to a SHIP counselor and determine whether SHIPs have the knowledge and expertise to correctly respond to questions about Medicare coverage.

## Methods

The Boston University Medical Campus institutional review board deemed this cross-sectional study to be nonhuman participants research and, as such, informed consent was not required. We followed Strengthening the Reporting of Observational Studies in Epidemiology (STROBE) reporting guideline for cross-sectional studies.^16^

### Script Development

We collaborated with the mystery shopping firm Second to None^17^ to develop a script to test the accuracy and completeness of information provided by SHIP counselors. We developed 2 scripts: one for shoppers posing as Medicare eligible only (eAppendix 1 in Supplement 1), and one for those posing as dual eligibles (eAppendix 2 in Supplement 1). Independent mystery shoppers aged 55 years and older were contracted to conduct the shops. They received the script for their assigned scenario along with detailed instructions and a questionnaire to capture information provided by SHIP counselors (eAppendix 3 in Supplement 1).

Our Medicare-only script tested knowledge of general Medicare coverage options, differences between Traditional Medicare (TM) and MA, and counselors’ ability to answer questions about specific MA plans. The dual-eligible script tested knowledge about integrated care options, Medicaid coverage of Medicare costs, and partial Medicaid benefits. Medicaid eligibility for people aged 65 years and older is determined based on income and asset criteria that varies by state^14^ and requires a more detailed analysis of an applicant’s finances than a SHIP counselor can provide. Therefore, questions were framed in terms of hypothetical situations (ie, “if I am still eligible for Medicaid”).

The shopping scripts, instructions, and questionnaire were reviewed by a panel of 5 researchers and analysts with expertise in Medicare and dual-eligible topics. The panel ensured that the scripts would test basic knowledge and that the elements of accurate and complete answers were reasonably characterized for each question. We piloted scripts at 10 SHIP sites in Indiana before finalization.

### Identification of Sites, MA Plans, and Clinicians for Mystery Shopping

To choose sites, we leveraged a SHIP site directory^16^ that includes information about likely in-person SHIP site locations and their associated zip code tabulation areas, including their geographic region, the median number of MA plans available (as proxy for the complexity of information a counselor might convey), and terciles of median household income. We selected 128 sites in 16 states representing diversity across each variable. We planned 2 phone shops at each site (1 per scenario) for a total of 256 phone shops. We planned 2 additional face-to-face shops at 20 of the 128 sites. After selecting sites, we used the CMS Plan Compare tool^18^ to identify specific plans that shoppers could reference and individual plan directories to identify primary care practitioners who were in-network, within 30 minutes and 20 miles from the SHIP site.

### Statistical Analysis

Secret shopping ran from September 2023 through August 2024. When sites on the original target list could not be reached after 2 attempts, we selected alternative sites with similar characteristics. This occurred for 7 sites. If sites on the list were reached but the script could not be completed, we recorded them as incomplete shops. Two analysts independently assessed shopping data to classify reasons for incomplete shops. We used questionnaire data to categorize counselor responses as accurate and complete, accurate but incomplete, not substantive, and incorrect. We scored accurate answers as accurate and complete if all components of a given question were substantively answered correctly, and accurate and incomplete if the counselor addressed only part of the question or excluded important details. For instance, we scored answers on the subject of timing for initial Medicare enrollment and subsequent changes as “accurate and complete” if the counselor: (1) mentioned that the shopper can enroll in Medicare and select coverage within 3 months of turning 65 years of age and (2) mentioned the open enrollment period, annual election period, or special enrollment periods in reference to when changes could be made. We coded answers as “substantive but incomplete” if some substantive information was provided but both components of the question were not answered (eg, if a counselor discussed initial enrollment upon turning age 65 but did not discuss when changes could be made) (eAppendix 4 in Supplement 1). If counselor responses did not fully align with the structure of the scoring system, 2 analysts reviewed and classified responses. Disagreements in classification were resolved through discussion. We used Stata 16 (StataCorp) to tabulate the frequency of shop completion and experiences, characteristics of completed shops, and response accuracy and completeness. We compared phone and face-to-face response accuracy with 2-sample *t* tests. *P* < .05 was considered statistically significant. Statistical analysis was performed from August to November 2024.

## Results

**Figure 1.**
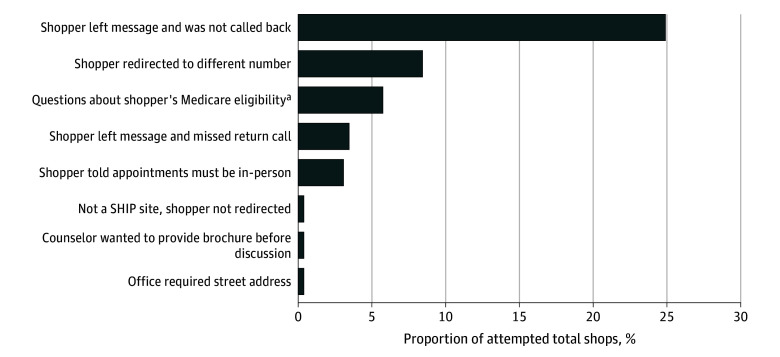
Failed Shops as a Share of All Attempted Phone Shops, by Reason for Failure SHIP indicates State Health Insurance Assistance Program. ^a^Shopper told to go to Social Security Office to see if they qualified for Medicare or to review Medicare website or to sign up for Medicare.

In total, 306 shops were attempted at 131 unique SHIP sites in 16 states. Of those, 184 (60.1%) were completed. All of the 45 attempted face-to-face shops were completed. Of 261 attempted phone shops, 139 (53.3%) were completed. The most common reason for an incomplete phone shop attempt was that the shopper left their information but did not receive a return call after 2 attempts within 2 business days (Figure 1).

Of 184 completed shops, 88 used the Medicare-only script and 96 used the dual-eligible script. Completed shops occurred at 96 unique sites in 16 states: 33 sites were shopped once, 43 were twice, 13 were 3 times, and 7 were 4 times. The mean (SD) meeting time was 35.8 (16.8) minutes for the Medicare-only scenario and 24.6 (13.6) minutes for the dual-eligible scenario (Table 1).

**Table 1.** Characteristics of Completed Shops

Characteristic	No. (%)		
	All shops (n = 184)	Phone (n = 139)	Face-to-face (n = 45)
Timeframe for shop			
Q4 2023	50 (27.2)	50 (36.0)	0
Q1 2024	35 (19.0)	32 (23.0)	3 (6.67)
Q2 2024	60 (32.6)	35 (25.2)	25 (55.6)
Q3 2024	39 (21.2)	22 (15.8)	17 (37.8)
Region where shop occurred			
Midwest	28 (15.2)	24 (17.3)	4 (8.9)
Northeast	56 (30.4)	39 (28.1)	17 (37.8)
South	42 (22.8)	29 (20.9)	13 (28.9)
West	58 (31.5)	47 (33.8)	11 (24.4)
Medicaid expansion state			
Yes	175 (95.1)	134 (96.4)	41 (91.1)
No	9 (4.9)	5 (3.6)	4 (8.9)
Meeting time, mean (SD) [range], min^a^			
Medicare-only scenario	35.8 (16.8) [9.0-113.0]	31.0 (11.8) [9.0-60.0]	49.3 (21.2) [16.0-113.0]
Dual-eligible scenario	24.6 (13.6) [4.5-70.0]	20.2 (8.9) [4.5-52.0]	39.3 (16.1) [9.35-70.0]

Abbreviation: Q, quarter.

^a^ Excludes 1 observation due to presumed data entry error.

### Accuracy and Completeness of Information Provided by Counselors

The accuracy and completeness of information provided to shoppers varied by topic. The mean (SD) percentage of accurate and complete answers was 40.0% (25.7%), and the mean (SD) percentage of accurate but incomplete responses was 34.3% (29.4%). The percentage of responses with accurate (complete or incomplete) answers ranged from 26.1% (23 of 88) to 94.3% (83 of 88). The mean (SD) percentage of nonsubstantive responses was 18.1% (13.8%). Overall, responses were unlikely to be inaccurate (mean [SD], 6.7% [5.4%]).

### Initial Enrollment in Medicare

Most counselors provided accurate and complete (57.1% [105 of 184]) or accurate but incomplete (33.2% [61 of 184]) information about when an individual soon to age into Medicare could enroll in Medicare coverage and change their coverage later (Table 2). More than half of counselors in the Medicare-only scenario (62.5% [55 of 88]) provided accurate and complete information about whether an individual with employer-sponsored coverage could maintain that coverage. However, 11.4% of counselors (10 of 88) incorrectly stated that the shopper must enroll or would be automatically enrolled in Medicare.

**Table 2.** Accuracy and Completeness of Information Provided by Counselors by Shop Scenario

Question and script scenario	No. (%)				
	Accurate and complete	Accurate but incomplete	Not substantive	Incorrect	Incomplete data^a^
Both scenarios (n = 184)					
Timing for initial Medicare enrollment and subsequent changes	105 (57.1)	61 (33.2)	13 (7.1)	5 (2.7)	0
Medicare-only scenario (n = 88)					
Medicare enrollment and interaction with employer plan	55 (62.5)	20 (22.7)	3 (3.4)	10 (11.4)	0
Differences between TM and MA	5 (5.7)	78 (88.6)	5 (5.7)	0 (0)	0
Medicare supplement plan considerations	3 (3.4)	78 (88.6)	6 (6.8)	1 (1.1)	0
Long-term care coverage	76 (86.4)	1 (1.1)	5 (5.7)	5 (5.7)	1
Prescription drug coverage	21 (23.9)	61 (69.3)	5 (5.7)	1 (1.1)	0
Whether specific PCP is in network for specific MA plan	23 (26.1)	0 (0)	57 (64.8)	8 (9.1)	0
Premium for specific MA plan	17 (19.3)	42 (47.7)	19 (21.6)	8 (9.1)	2 (2.3)
Whether specific MA plan allows out-of-network care	54 (61.4)	14 (15.9)	13 (14.8)	4 (4.5)	3 (3.4)
In-network PCP copay	43 (48.9)	6 (6.8)	23 (26.1)	13 (14.8)	3 (3.4)
Maximum out-of-pocket limit	41 (46.6)	10 (11.4)	25 (28.4)	12 (13.6)	0
Whether specific MA plan includes coverage for prescription drugs	67 (76.1)	2 (2.3)	14 (15.9)	2 (2.3)	3 (3.4)
Whether specific MA plan covers specific drug (Lipitor) and/or its generic equivalent	40 (45.5)	15 (17.0)	28 (31.8)	3 (3.4)	2 (2.3)
Dual-eligible scenario (n = 96)					
Options for enrolling in Medicare with full Medicaid benefits	23 (24.0)	46 (47.9)	19 (19.8)	8 (8.3)	0
Considerations for choosing an integrated care option (D-SNP)	1 (1.0)	71 (74.0)	23 (24.0)	1 (1.0)	0
D-SNP availability in shopper's area	66 (68.8)	9 (9.4)	16 (16.7)	5 (5.2)	0
Coverage for long-term care	10 (10.4)	57 (59.4)	9 (9.4)	20 (20.8)	0
Medicaid coverage of Medicare premiums and cost sharing	63 (65.6)	10 (10.4)	18 (18.8)	4 (4.2)	1 (1.0)
Medicare cost sharing assistance	26 (27.1)	45 (46.9)	17 (17.7)	8 (8.3)	0

Abbreviations: D-SNP, dual-eligible special needs plan; MA, Medicare Advantage; PCP, primary care practitioner; TM, Traditional Medicare.

^a^ Shopper did not or could not ask all component questions considered in scoring of responses.

### Considerations for Choosing Among Medicare Coverage Options

Most of the counselors in the Medicare-only scenario (94.3% [83 of 88]) provided at least some accurate information on the differences between TM and MA, although few (5.7% [5 of 88]) provided a complete response (Table 2). More than half of counselors correctly noted that MA offers additional benefits such as vision or dental (65.9% [58 of 88]) and may have lower premiums (60.2% [53 of 88]), but offer more restrictive coverage of clinicians than TM (64.8% [57 of 88]). Most counselors (93.2% [82 of 88]) provided at least some accurate information on Medicare Part D coverage.

### Ability to Answer Questions About a Particular Health Plan

When asked about a specific health plan and whether a specific physician was in network, approximately one-quarter of counselors in the Medicare-only scenario (26.1% [23 of 88]) reviewed the health plan directory and provided an accurate and complete answer. Approximately two-thirds of counselors (64.8% [57 of 88]) provided an answer that was not substantive (ie, by saying that the shopper would need to contact the plan). Although two-thirds of counselors (67.0% [59 of 88]) provided accurate information about the health plan’s premium, a smaller portion (19.3% [17 of 88]) clarified that the Part B premium would also apply.

More than three-quarters of counselors (76.1% [67 of 88]) provided accurate and complete information about whether the health plan covered prescription drugs. Although most counselors (62.5% [55 of 88]) reviewed the formulary to determine correctly that either Lipitor (Viatris) or its generic equivalent atorvastatin would be covered, fewer than half (45.5% [40 of 88]) provided complete information—even after a follow-up question—to clarify that the generic option would be covered at a lower out-of-pocket cost than the brand name drug.

### Interactions Between Medicare and Medicaid and Key Issues for Dual Eligibility

Most counselors in the dual-eligible scenario (65.6% [63 of 96]) accurately and completely explained that if the shopper remained fully eligible for Medicaid upon turning age 65, Medicaid would cover applicable cost sharing regardless of the Medicare coverage option chosen. Only one-quarter of counselors (27.1% [26 of 96]) accurately and completely responded to a question about Medicare cost sharing for individuals who are not eligible for full Medicaid benefits by referring enrollees to both an MSP and to the LIS.

Few counselors in the dual-eligible scenario (10.4% [10 of 96]) accurately and completely explained that Medicaid, not Medicare, would cover long-term care if the shopper met additional criteria related to resources and medical or functional need. One-fifth of counselors (20.8% [20 of 96]) provided incorrect information, suggesting Medicare covered long-term care or stating that Medicaid did not cover long-term care.

### Navigating Integrated Options for Dual Eligibles

When shoppers asked about available coverage options if they remain eligible for Medicaid after aging into Medicare, fewer than half of counselors in the dual-eligible scenario (44.8% [43 of 96]) mentioned D-SNPs (eTable 1 in Supplement 1). However, once asked directly whether D-SNPs were available in the shopper’s service area, approximately two-thirds of counselors (68.8% [66 of 96]) correctly responded (Table 2).

**Figure 2.**
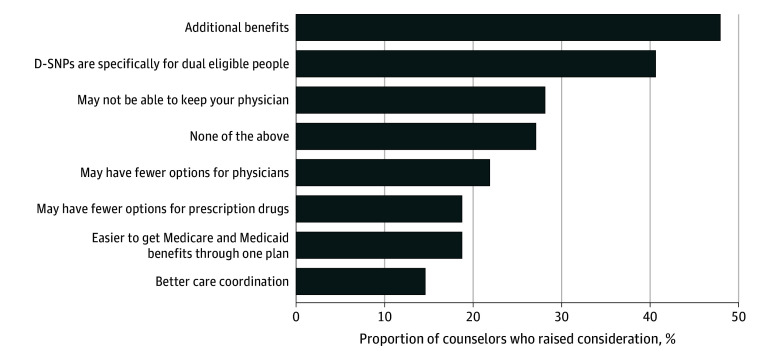
Share of Counselors in the Dual-Eligible Scenario Who Raised Specific Considerations for Choosing a Dual-Eligible Special Needs Plan (D-SNP)

When asked specifically about considerations for D-SNPs, 70 counselors (72.9%) offered at least 1 key characteristic, whereas 20 counselors (21.8%) offered 5 or more (eTable 1 in Supplement 1). The most common characteristics mentioned were that D-SNPs offer additional benefits (n = 46) and are tailored to dual eligibles (n = 39) (Figure 2). By contrast, few counselors mentioned that D-SNPs offer better care coordination (n = 14).

### Accuracy and Completeness by Phone vs Face-to-Face

The mean (SD) percentage of accurate and complete answers was higher with face-to-face shops (51.8% [30.5%]) than with phone shops (36.0% [25.1%]) (*P* < .001). For face-to-face shops, the percentage of responses with accurate (complete or not) answers ranged from 34.8% (8 of 23) to 100% (eTables 2 and 3 in Supplement 1). For phone shops, the percentage of responses with accurate (complete or not) answers ranged from 23.1% (15 of 65) to 92.3% (60 of 65). The mean (SD) percentage of responses that did not substantively answer the question was lower in face-to-face shops (8.9% [11.8%]) than phone shops (21.2% [15.3%]) (*P* < .001). There was no statistically significant difference in the mean (SD) percentage of responses that were incorrect across mode of shop (face-to-face: 5.1% [6.5] vs phone: 7.1% [6.0%]; *P* = .06).

### Evidence of Bias

While our script did not allow us to systematically assess counselor bias, a review of free-text commentary from shoppers indicated that bias was present in several encounters. For example, one shopper noted: “The counselor told me that Medicare Supplement Plans were expensive and complicated. She simply said that she doesn't recommend them.”

## Discussion

In this cross-sectional, mystery shopping study of SHIP sites across the nation, we characterized experiences contacting SHIP sites and the quality of information provided. Challenges reaching SHIP sites indicate possible limitations in the ability to meet demand for services. Responses to mystery shoppers suggest that SHIP counselors may be best prepared to provide accurate and complete information on topics including initial Medicare enrollment and offering considerations for MA, TM, Part D coverage, and coverage of specific medications. However, counselors may be less well-prepared or willing to address considerations for enrollment in D-SNPs, discuss eligibility for Medicaid-covered long-term care, or answer questions about specific MA plans, particularly those related to network membership of clinicians (perhaps because MA plan directories are not currently shown within the Plan Finder tool and require navigating through a link to an external site).^18^

Our findings also suggest that beneficiaries may be more likely to receive accurate and complete information via face-to-face interactions than phone interactions. Future research on this topic may be helpful given the findings of prior research that indicate differential access to in-person SHIP sites.^3^

Our findings complement those of other studies that have highlighted potential needs for improved training of SHIP counselors.^3,10,15^ Notably, SHIP counselors previously expressed a desire for more training in topics relevant to dual eligibles,^15^ which is reflected by variable quality in responses about dual-eligible coverage decisions in the current study. To better meet the needs of beneficiaries, state SHIP programs may wish to leverage the national SHIP technical assistance center’s trainings on Medicaid and dual eligibility if they are not yet doing so. There may also be opportunities to expand the national curriculum offered to reflect increasing complexity in coverage options. However, states may need to design and provide additional trainings to cover state-specific coverage considerations for dual eligibles.

Enrollment in MA, including D-SNPs,^1^ is increasing, and the number of coverage options continues to increase. Recent changes to CMS regulations call for third-party marketers of MA products to mention SHIP as a federal resource that can assist with coverage decisions. Moreover, CMS has engaged in efforts to promote integrated plans for dual eligibles. If these efforts are successful, SHIP counselors may encounter an increasing volume of requests for services and questions about MA plans and D-SNPs. Our findings highlight opportunities to improve education and training to address counselors’ knowledge gaps and ensure Medicare beneficiaries receive coverage that meets their needs. The SHIP program may benefit from additional federal funding to expand availability of SHIP services, in part through hiring additional counselors, to better meet demand. It may also benefit from enhanced development and provision of training and informational resources to ensure counselors can discuss characteristics of specific plans such as D-SNPs and how they relate to beneficiaries’ circumstances.

### Limitations

This study has limitations. First, mystery shopping encounters reflect the experience of a beneficiary who knows about and can reach SHIP services, knows Medicare terminology, and can communicate well in English. However, even in these scenarios, we encountered challenges in reaching SHIP counselors and obtaining complete information. Second, scoring relied on shoppers’ ability to categorize a counselor’s answer into the choice that best described the answer (eAppendix 3 in Supplement 1). Consequently, shoppers may have selected an answer that corresponded with the “accurate” designation even if the answer contained minor incorrect elements. As such, our results for the number of inaccurate answers are conservative. Similarly, evaluating accuracy and completeness relied on publicly available information about each selected plan and available clinicians, which itself is often flawed.^19,20^ Thus, an additional limitation is that the elements of an accurate and complete answer as defined by researchers may not equate to the elements that would adequately assist a beneficiary in selecting coverage that best meets their needs. For example, when posed with a question regarding a particular clinician’s network participation, a counselor who directed a shopper to contact the plan would have received a score of “not substantive.” However, in practice, this answer may be more helpful to the beneficiary than an “accurate and complete” answer that confirmed a clinician’s network status.^16,17^ Additionally, because a SHIP site’s address does not necessarily reflect where counselors provide services, we were precluded from comparing differences in accuracy across site characteristics. Further study of such differences may be helpful in directing resources more effectively. Despite these limitations, to our knowledge, this is the most in-depth assessment of the quality of information provided by SHIP sites to date.

## Conclusions

In this cross-sectional, mystery shopping study, shoppers posing as Medicare beneficiaries encountered challenges in reaching SHIP sites and wide variation in the accuracy and completeness of information about Medicare coverage options. Given the growth in MA and efforts by CMS and others to counter deceptive marketing practices and bias from agents or brokers, in part by directing beneficiaries to SHIPs, policymakers should consider providing the SHIP program with additional funding to expand the capacity of individual SHIP sites and to provide counselors with additional training and materials in priority areas including dual-eligible topics.

## Notes

Supplement 1. **eTable 1.** Information Provided Regarding Dual-Eligible Special Needs Plans, by Shop Modality **eTable 2.** Accuracy and Completeness Summary Score, Face-to-Face Shops **eTable 3.** Accuracy and Completeness Summary Score, Phone Shops **eAppendix 1.** Mystery Shopper Script for Medicare-Only Scenarios **eAppendix 2.** Mystery Shopper Script for Dual-Eligible Scenarios **eAppendix 3.** Shopper Instructions and Questionnaire **eAppendix 4.** Scoring Guide

Supplement 2.
Data Sharing Statement
