# Supplementary material for: Accuracy of Medicare Information Provided by State Health Insurance Assistance Programs
Source: JAMA Netw Open. 2025 Apr 1;8(4):e252834. doi: 10.1001/jamanetworkopen.2025.2834 (PMC11962663; doi:10.1001/jamanetworkopen.2025.2834)
Supplement: Supplement 1. — eTable 1. Information Provided Regarding Dual-Eligible Special Needs Plans, by Shop Modality eTable 2. Accuracy and Completeness Summary Score, Face-to-Face Shops eTable 3. Accuracy and Completeness Summary Score, Phone Shops eAppendix 1. Mystery Shopper Script for Medicare-Only Scenarios eAppendix 2. Mystery Shopper Script for Dual-Eligible Scenarios eAppendix 3. Shopper Instructions and Questionnaire eAppendix 4. Scoring Guide [file jamanetwopen-e252834-s001.pdf]

Supplemental Online Content

Dugan K, Peterson I, Dorneo A, Garrido MM. Accuracy of Medicare information provided by State Health Insurance Assistance Programs. *JAMA Netw Open.* 2025;8(4):e252834. doi:10.1001/jamanetworkopen.2025.2834

eTable 1. Information Provided Regarding Dual-Eligible Special Needs Plans, by Shop Modality

eTable 2. Accuracy and Completeness Summary Score, Face-to-Face Shops

eTable 3. Accuracy and Completeness Summary Score, Phone Shops

eAppendix 1. Mystery Shopper Script for Medicare-Only Scenarios

eAppendix 2. Mystery Shopper Script for Dual-Eligible Scenarios

eAppendix 3. Shopper Instructions and Questionnaire

eAppendix 4. Scoring Guide

This supplemental material has been provided by the authors to give readers additional information about their work.

eTable 1. Information Provided Regarding Dual Eligible Special Needs Plans, by Shop Modality

	All Shops (n=96)	Phone (n=45)	Face-to-Face (n=22)
**Mentioned D-SNPs in Answer to General Question on Dual-Eligible Coverage Options**			
Yes	43 (44.8)	31 (41.9)	12 (54.5)
No	53 (55.2)	43 (58.1)	10 (45.5)
**Specific Considerations Mentioned for Choosing a D-SNP vs Other Options**			
Additional benefits	46 (47.9)	30 (40.5)	16 (72.7)
Better care coordination	14 (14.6)	8 (10.8)	6 (27.3)
Makes it easier to get Medicare and Medicaid benefits through one plan	18 (18.8)	11 (14.9)	7 (31.8)
Dual eligible plans are specifically made for dual eligible people	39 (40.6)	29 (39.2)	10 (45.4)
You may not be able to keep your doctor	27 (28.1)	18 (24.3)	9 (40.9)
You may have fewer options for doctors to choose from than if you choose TM	21 (21.9)	19 (25.7)	2 (9.1)
You may have fewer options for prescription drugs than if you choose TM	18 (18.8)	8 (10.8)	10 (45.5)
None of the above	26 (27.1)	23 (31.1)	3 (13.6)
**Total Considerations Mentioned**			
0	26 (27.1)	23 (31.1)	3 (13.6)
1 to 2	17 (17.7)	15 (20.3)	2 (9.1)
3 to 4	24 (25.0)	18 (24.3)	6 (27.3)
5 to 6	12 (12.5)	8 (10.8)	4 (18.2)
7	8 (8.3)	6 (8.1)	2 (9.1)

D-SNP = Dual-Eligible Special Needs Plan

TM = Traditional Medicare

eTable 2. Accuracy and Completeness Summary Score, Face-to-Face Shops

Question and Script Scenario	Accurate and Complete (% of shops)	Accurate but Incomplete (% of shops)	Not Substantive (% of shops)	Incorrect (% of shops)	Incomplete Data[a] (% of shops)
Both Scenarios (N=45)					
Timing for Initial Medicare Enrollment & Subsequent Changes	37 (82.2)	7 (15.6)	1 (2.2)	0 (0)	0 (0)
Medicare-Only Scenario (n=23)					
Medicare Enrollment & Interaction with Employer Plan	14 (60.9)	8 (34.8)	1 (4.3)	0 (0)	0 (0)
Differences between Traditional Medicare and Medicare Advantage	4 (17.4)	19 (82.6)	0 (0)	0 (0)	0 (0)
Medicare Supplement Plan Considerations	1 (4.3)	22 (95.7)	0 (0)	0 (0)	0 (0)
Long-Term Care Coverage	21 (91.3)	0 (0)	2 (8.7)	0 (0)	0 (0)
Prescription Drug Coverage Considerations	8 (34.8)	15 (65.2)	0 (0)	0 (0)	0 (0)
Whether Specific PCP is in Network for Specific Plan	8 (34.8)	0 (0)	12 (52.2)	3 (13)	0 (0)
Premium for Specific Plan	10 (43.5)	11 (47.8)	1 (4.3)	1 (4.3)	0 (0)
Whether Specific Plan Allows Out-of-Network Care	15 (65.2)	5 (21.7)	1 (4.3)	2 (8.7)	0 (0)
In-Network PCP Copay	15 (65.2)	1 (4.3)	3 (13)	4 (17.4)	0 (0)
Maximum Out-of-Pocket Limit	20 (87)	1 (4.3)	2 (8.7)	0 (0)	0 (0)
Whether Specific Plan Includes Coverage for Prescription Drugs	21 (91.3)	1 (4.3)	1 (4.3)	0 (0)	0 (0)
Whether Specific Plan Covers Specific Drug (Lipitor) and/or its Generic Equivalent	15 (65.2)	5 (21.7)	2 (8.7)	1 (4.3)	0 (0)
Dual-Eligible Scenario (n=22)					
Options for Enrolling in Medicare with Full Medicaid Benefits	9 (40.9)	8 (36.4)	3 (13.6)	2 (9.1)	0 (0)
Considerations for Choosing an Integrated Care Option (D-SNP)*	0 (0)	19 (86.4)	2 (9.1)	1 (4.5)	0 (0)
D-SNP Availability in Shopper's Area	18 (81.8)	1 (4.5)	3 (13.6)	0 (0)	0 (0)
Coverage for Long-Term Care	1 (4.5)	16 (72.7)	0 (0)	5 (22.7)	0 (0)
Medicaid Coverage of Medicare Premiums and Cost Sharing	19 (86.4)	2 (9.1)	0 (0)	1 (4.5)	0 (0)
Medicare Cost Sharing Assistance	6 (27.3)	9 (40.9)	5 (22.7)	2 (9.1)	0 (0)

D-SNP = Dual-Eligible Special Needs Plan

a) Shopper did not or could not answer all component questions considered in scoring of responses.

© 2025 Dugan K et al. *JAMA Network Open*

eTable 3. Accuracy and Completeness Summary Score, Phone Shops

Question and Script Scenario	Accurate and Complete (% of shops)	Accurate but Incomplete (% of shops)	Not Substantive (% of shops)	Incorrect (% of shops)	Incomplete Data[a] (% of shops)
Both Scenarios (N=139)					
Timing for Initial Medicare Enrollment & Subsequent Changes	68 (48.9)	54 (38.8)	12 (8.6)	5 (3.6)	0 (0)
Medicare-Only Scenario (n=65)					
Medicare Enrollment & Interaction with Employer Plan	41 (63.1)	12 (18.5)	2 (3.1)	10 (15.4)	0 (0)
Differences between Traditional Medicare and Medicare Advantage	1 (1.5)	59 (90.8)	5 (7.7)	0 (0)	0 (0)
Medicare Supplement Plan Considerations	2 (3.1)	56 (86.2)	6 (9.2)	1 (1.5)	0 (0)
Long-Term Care Coverage	55 (84.6)	1 (1.5)	3 (4.6)	5 (7.7)	1 (1.5)
Prescription Drug Coverage Considerations	13 (20)	46 (70.8)	5 (7.7)	1 (1.5)	0 (0)
Whether Specific PCP is in Network for Specific Plan	15 (23.1)	0 (0)	45 (69.2)	5 (7.7)	0 (0)
Premium for Specific Plan	7 (10.8)	31 (47.7)	18 (27.7)	7 (10.8)	2 (3.1)
Whether Specific Plan Allows Out-of-Network Care	39 (60)	9 (13.8)	12 (18.5)	2 (3.1)	3 (4.6)
In-Network PCP Copay	28 (43.1)	5 (7.7)	20 (30.8)	9 (13.8)	3 (4.6)
Maximum Out-of-Pocket Limit	21 (32.3)	9 (13.8)	23 (35.4)	12 (18.5)	0 (0)
Whether Specific Plan Includes Coverage for Prescription Drugs	46 (70.8)	2 (3.1)	13 (20)	1 (1.5)	3 (4.6)
Whether Specific Plan Covers Specific Drug (Lipitor) and/or its Generic Equivalent	25 (38.5)	10 (15.4)	26 (40)	2 (3.1)	2 (3.1)
Dual-Eligible Scenario (n=74)					
Options for Enrolling in Medicare with Full Medicaid Benefits	14 (18.9)	38 (51.4)	16 (21.6)	6 (8.1)	0 (0)
Considerations for Choosing an Integrated Care Option (D-SNP)	1 (1.4)	52 (70.3)	21 (28.4)	0 (0)	0 (0)
D-SNP Availability in Shopper's Area	48 (64.9)	8 (10.8)	13 (17.6)	5 (6.8)	0 (0)
Coverage for Long-Term Care	9 (12.2)	41 (55.4)	9 (12.2)	15 (20.3)	0 (0)
Medicaid Coverage of Medicare Premiums and Cost Sharing	44 (59.5)	8 (10.8)	18 (24.3)	3 (4.1)	0 (0)
Medicare Cost Sharing Assistance	20 (27)	36 (48.6)	12 (16.2)	6 (8.1)	0

D-SNP = Dual-Eligible Special Needs Plan

a) Shopper did not or could not answer all component questions considered in scoring of responses.

© 2025 Dugan K et al. *JAMA Network Open*

eAppendix 1. Mystery Shopper Script for Medicare-only Scenarios

Script – Medicare Eligible Only Scenario

Important! Make sure your shop confirmation says Scenario 2: Medicare Eligible Only.

Tip! Before calling, write down the doctor's name, the health plan's name, and the city, state, zip code, and county from your shop confirmation so you don't sound like you are looking up the information.

Your Scenario: You must role-play to be turning 65 in a couple of months, which makes you Medicare eligible (known as 'aging-in').

- Start the conversation by making the Opening Statement listed below.
- Answer questions based on the Scenario Details listed below.
- Ask all Required Questions listed below in the order they are listed.
  - Your primary objective is to ask every question listed. Do not skip any questions.
  - If the counselor indicates they don't know the answer to a question or directs you to another resource for an answer, move on to the next question until all questions have been asked.

Opening Statement: Make the following statement (exactly or similarly).

- I am turning 65 soon and will be eligible for Medicare. I would like help choosing my coverage. Can you help?

Scenario Details: Present yourself as follows when answering questions.

- You live in the city, state, zip code, and county listed in your shop confirmation.
- You are not on Medicaid (state assistance) and do not qualify for it.
- You are just becoming eligible for Medicare and will be 65 within the next few months.
  - If asked for your birthdate, give a date 2 months out, but the year must be 1958 or 1959 (depending on date of shop).
  - If asked, you have received your Medicare card in the mail, but you don't have it with you.
- You currently have employer-based health coverage that includes coverage for prescription drugs.
  - If asked how much you pay for prescriptions, say you generally pay just a few dollars for most prescriptions and about $50 for more expensive medications.
  - The only medication you take is **Lipitor** (10mg daily for cholesterol).
- You are relatively healthy and see a physician a few times a year, usually just for checkups or minor problems.
  - If asked for the name of your primary care physician, give the name of the doctor listed in your shop confirmation.

Required Questions: Ask all questions in order exactly as written, filling in blanks from your shop confirmation as directed. Do not alter the wording otherwise. Take detailed notes so you can document the full response to each question in your report.

- **Question #1:** When can I select my Medicare coverage plan, and when can I change it later?
- **Question #2:** Do I have to sign up for Medicare when I turn 65, or can I remain on my employer plan?
- **Question #3:** I keep hearing about Medicare Advantage. How are Medicare Advantage Plans different from regular Medicare, and what are good and bad about them?
- **Question #4:** What is a Medicare Supplement Plan, how much do they cost, and when do I enroll?
- **Question #5:** Do either regular Medicare, Medicare Advantage Plans, or Medicare Supplement Plans cover long-term care?
- **Question #6:** What are my options for Medicare prescription drug coverage, and how should I choose?
- **Question #7:** My friend told me about [plan name listed in your shop confirmation]. Can you tell me if [name of doctor listed in your shop confirmation] is in the network there?
- **Question #8:** What is the monthly premium for [plan name listed in your shop confirmation]?
- **Question #9:** Would [plan name listed in your shop confirmation] let me go out of network?
- **Question #10:** What is the copay for a primary care doctor visit for [plan name listed in your shop confirmation]?
  - **Note:** If both in-network and out-of-network copays are provided, write both down. If only one copay is given, ask if it's for in-network or out-of-network visits.
- **Question #11:** What is the copay to see a specialist for [plan name listed in your shop confirmation]?
  - **Note:** If both in-network and out-of-network copays are provided, write both down. If only one copay is given, ask if it's for in-network or out-of-network visits.
- **Question #12:** For [plan name listed in your shop confirmation], is there a limit on out-of-pocket costs I would have to

pay each year?

- **Question #13:** Does [plan name listed in your shop confirmation] include prescription drug coverage?
- **Question #14a:** I take Lipitor. Is that covered by [plan name listed in your shop confirmation]?
    - **Follow-up question #14b:** If the generic version of Lipitor is not mentioned, you must also ask, “Is a generic version of Lipitor covered? I would be willing to take that.”
- **Question #15:** How much would Lipitor be out of pocket vs. the generic version?
    - **Note:** If the counselor mentions it varies by pharmacy, say you are willing to go to a local pharmacy that is in network or offers the cheapest drugs. You do not want to fill prescriptions by mail.
- **Question #16:** I have a friend who does not speak much English. Do you offer Spanish translation services at your location?

© 2025 Dugan K et al. *JAMA Network Open*

eAppendix 2. Mystery Shopper Script for Dual-Eligible Scenarios

Script – Dual Eligible Scenario (On Medicaid and Eligible for Medicare)

Important! Make sure your shop confirmation says Scenario 1: Dual Eligible.

Tip! Before calling, write down the doctor's name and the city, state, zip code and county from your shop confirmation so you don't sound like you are looking up the information.

Your Scenario: You must role-play to be on Medicaid (state assistance) AND turning 65 in a couple of months, which makes you Medicare eligible (known as 'aging-in'). People who are on Medicaid AND eligible for Medicare are known as 'Dual Eligible.'

- Start the conversation by making the Opening Statement listed below.
- Answer questions based on the Scenario Details listed below.
- Ask all Required Questions listed below in the order they are listed.
  - Your primary objective is to ask every question listed. Do not skip any questions.
  - If the counselor indicates they don't know the answer to a question or directs you to another resource for an answer, move on to the next question until all questions have been asked.

Opening Statement: Make the following statement (exactly or similarly).

- I am turning 65 soon. I have Medicaid right now, and I expect to be able to keep it when I turn 65. I'm waiting to hear if I will be able to keep it. I have some questions about this, and I'm hoping you can help with that.

Scenario Details: Present yourself as follows when answering questions.

- You live in the city, state, zip code, and county listed in your shop confirmation.
- You have an income of about $1,600, and it is just you in your household.
- You are on Medicaid (state assistance) right now.
  - If asked for your Medicaid card number, say you do not have it with you.
  - If asked if you are enrolled in a specific Medicaid health plan, say yes, but you do not recall which one it is.
- You are just becoming eligible for Medicare and are turning 65 within a few months.
  - If asked for your birthdate, give a date 2 months out, but the year must be 1958.
  - If asked, you have received your Medicare card in the mail, but you don't have it with you.
- You currently have prescription drug coverage at no cost to you through Medicaid.
  - If asked how much you pay for prescriptions, say it's typically just a few dollars.
  - The only medication you take is **Lipitor** (10mg daily for cholesterol).
- You are relatively healthy and see a physician a few times a year, usually just for checkups or minor problems.
  - If asked for the name of your primary care physician, give the name of the doctor listed in your shop confirmation.

Required Questions: Ask all questions in order exactly as written. Do not alter the wording. Take detailed notes so you can document the full response to each question in your report.

- **Question #1:** When can I select my Medicare coverage plan, and when can I change it later?
- **Question #2:** If I stay eligible for Medicaid, what are my options for enrolling in Medicare?
- **Question #3:** I saw a commercial about special plans for people with both Medicaid and Medicare. Can you tell me more about them and why I may or may not want to choose one?
- **Question #4:** Are special plans for people with Medicaid and Medicare available in my area?
  - **Note:** Give the city, state, zip code, and county listed in your shop confirmation.
- **Question #5a:** If I ever need long-term care in the future, would it be covered under any of the Medicare options we've talked about?
  - **Follow up question #5b:** What about Medicaid? If I stay eligible, will Medicaid pay for long-term care?
- **Question #6:** If I stay eligible for Medicaid, will it pay for my Medicare premiums and cost sharing and deductibles no matter if I choose a Dual Eligible Special Needs Plan, a Medicare Advantage Plan, or regular Medicare?
- **Question #7:** I was told that if I'm not eligible for Medicaid, there are other programs to help with out-of-pocket costs for Medicare. My income is $1,600 a month. Would I get any help?
- **Question #8:** I have a friend who does not speak much English. Do you offer Spanish translation services at your location?

eAppendix3. Shopper Instructions and Questionnaire

SHIP Insurance study
knowledge & accessibility

Instructions
•
questionnaire

This is a research study. Call a State Health Insurance Assistance Program (SHIP) to present your assigned scenario and ask all assigned knowledge questions, documenting responses completely. Calls must be recorded through CX Group's IVR system (excludes locations in California, Massachusetts, New Hampshire, and Washington). The call is expected to take 30-45 minutes.

Before You Begin

- Review your shop confirmation
- Be familiar with your assigned scenario
- Know the opening statement to make
- Have the list of knowledge questions

Don't Forget!

- Record the call using IVR (if applicable)
- Make the opening statement
- Follow your assigned scenario
- Ask all required knowledge questions

General Requirements

- You must be 55 or older to participate and able to role-play as someone who is turning 65.
- Read all instructions and the entire questionnaire before you complete the shop.
- Click here to review a summary of original Medicare, Medicare Advantage Plans, and Medicare Supplement Plans if you are not already familiar with them.
- You will be assigned 1 of 2 scenarios:
  - **Scenario 1:** Dual Eligible OR **Scenario 2:** Medicare Eligible Only
- Check your shop confirmation for the following information:
  - ✓ The city, state, zip code, and county to say you live in
  - ✓ Your assigned scenario
  - ✓ The name of the doctor to say is your primary care physician
  - ✓ The health plan name to ask about (Medicare Eligible Only scenario)
- Call Monday-Thursday only during the business hours listed in your shop confirmation. Do not call Friday, Saturday, or Sunday.
- Record the call by dialing into CX Group's IVR phone system.
- Submit your report to <web link> within 12 hours of completing the shop.
- Take detailed notes, and retain them for six months following your shop.
- You or your immediate family members cannot have ever worked for a SHIP office.

Shop Instructions

Step #1: Prepare for the call by becoming familiar with your assigned scenario and questions

- Click the link that matches your scenario, which can be found in your shop confirmation.
  - Click here to review instructions for Scenario 1: Dual Eligible.
  - Click here to review instructions for Scenario 2: Medicare Eligible Only.

Step #2: Call into CX Group's IVR phone system to record the conversation

- Call into CX Group's IVR phone system.
  - If your assigned location is in California, Massachusetts, New Hampshire, or Washington, do not record.
- Once dialed into the IVR system, you will be prompted to enter your assignment number. This can be found on your shop confirmation. Only enter the first series of numbers. For example, if your assignment number is 2089568-3658147, you would enter 2089568.
- Next, the system will ask you to dial the phone number you would like to call. Dial the SHIP office phone number found in your shop confirmation, and follow the prompts.
  - Do not dial a "1" before the area code once in the IVR system.
- If you get an error message from the IVR when calling the 866 phone number, try calling the alternate phone number for the IVR.
- You will get an IVR confirmation number. Write down the number, and enter it in your report.
- If more than one call to the SHIP office is required, all calls must be made through the IVR.

Step #3: If required, wait for a return call or make additional calls to connect with a counselor

- If you get voicemail or asked to leave your contact info, provide your name and phone number.
  - Wait 1 full business day for a return call. If you do not get a call back by the next business day, call again during allowed call hours and leave your contact info again if necessary.
  - Wait 1 more full business day for a return call. If you do not get a call back after the second call attempt, submit your report.
- If you are required to make an appointment, ask if you can make an appointment to speak with a counselor by phone (not in person).
  - Request to speak with someone by phone within 3 days. If a phone appointment is not available within 3 days, accept the next available date. Notify your scheduler of the date.
  - Be available and ready for the phone appointment at the agreed upon date/time. If the counselor misses the appointment, make at least one more call attempt during allowed call hours before submitting your report.
- When a counselor returns your call, do not answer. Send the call to voicemail, and immediately call the counselor back after dialing into CX Group's IVR phone system.
  - If you accidentally answer, ask for their direct number to call them right back.
- **Unsuccessful calls:** If you cannot complete the call on the first attempt because the office is closed, no one answers, or you receive a busy signal, make at least one more call attempt before submitting your report. Your second attempt must be on a different day of the week during allowed call hours.

Step #4: Speak with a SHIP counselor, and ask all required knowledge questions

- First, make the opening statement when the call is answered and when connected to a counselor (if you speak to another associate initially).
  - The opening statement can be found in your scenario document.
- Next, begin asking your assigned knowledge questions.
  - Ask the questions in the order listed in your scenario document.
  - Ask the questions exactly as they are worded in your scenario document.
  - Ask all questions in your scenario document. Do not skip any questions for any reason.
  - Document the counselor's response to each question by taking detailed notes.
- To identify plan information, the counselor should request a city, state, zip code, and/or county. Give them the information found in your shop confirmation.
- Let the counselor ask questions and provide information they feel is necessary. Do not interrupt them or cut the call short.
  - Respond to the counselor's questions using the info found in your scenario document.
  - Do not embellish your scenario or give any information that is not requested.
- Get the counselor's first name (and last name, if possible).
- If the call is disconnected at any point, call back immediately to resume the conversation with the same counselor.

QUESTIONNAIRE

Location:

Shop date:

Start time:

End time:

Connecting to SHIP Counselor

1. **Which scenario did you use?**
Select N/A only if you never spoke with a counselor (i.e., you were unable to ask any of your required questions).
[ ] Dual Eligible
[ ] Medicare Eligible Only
[ ] N/A – I could not reach a counselor after two attempts

2. **What date and time did you first call the SHIP office?**
Required format: MM/DD/YYYY HH:MM AM/PM

3. **What day of the week did you first call the SHIP office?**
[ ] Monday
[ ] Tuesday
[ ] Wednesday
[ ] Thursday
[ ] Friday
[ ] Saturday
[ ] Sunday
[ ] N/A – I could not reach a counselor after two attempts

4. **If you had to wait for a call back before you could speak to a counselor who could answer your questions, which of the following best fits your experience?**
[ ] I had to leave a voicemail and wait for a call back
[ ] A person collected my contact information and I had to wait for a call back
[ ] I had to submit my contact information online and wait for a call back
[ ] N/A – I did not have to wait for a call back
[ ] N/A – I could not reach a counselor after two attempts

5. **Date and time you left your first voicemail (or your contact information):**
If you could not speak to a counselor without waiting for a call back, enter the date and time you either left a voicemail or you left your contact information. Required format: MM/DD/YYYY HH:MM AM/PM

6. **If you had to wait for a call back, how long did it take for a counselor to contact you?**
[ ] Same day
[ ] Next business day
[ ] 2 business days
[ ] 3 business days
[ ] More than 3 business days
[ ] I was not contacted and had to call again
[ ] N/A – I did not have to wait for a call back
[ ] N/A – I could not reach a counselor after two attempts

7. **Date and time you spoke with a counselor to ask your questions:**
If you had to wait for a call back to speak with a counselor, this may be when your call was returned or when you called back after not receiving a return call. Required format: MM/DD/YYYY HH:MM AM/PM

8. **Name of the counselor you spoke to when asking your questions:**

9. **How would you rate the level of effort it took to connect with a counselor to ask your questions?**
[ ] 5 – Very easy
[ ] 4 – Easy
[ ] 3 – Neither easy nor difficult
[ ] 2 – Difficult
[ ] 1 – Very difficult
[ ] N/A – I could not reach a counselor after two attempts

10. **Please explain your rating:**

11. **Were you disconnected at any time after being connected to a counselor to ask your questions?**
[ ] Yes
[ ] No
[ ] N/A – I could not reach a counselor after two attempts

12. **If yes, describe what happened and how you reconnected with a counselor:**

13. What was the total time the counselor spent on the phone with you answering your questions?
Enter time in minutes:seconds (00:00) format.

14. In response to "When can I select my Medicare coverage plan, and when can I change it later?", did the counselor mention you can select Medicare coverage within 3 months of turning 65?
[ ] Yes
[ ] No
[ ] N/A – I could not reach a counselor after two attempts

15. In response to "When can I select my Medicare coverage plan, and when can I change it later?", did the counselor mention the Open Enrollment Period (10/15-12/7)?
[ ] Yes, mentioned Open Enrollment and provided these dates
[ ] Mentioned Open Enrollment but did not provide these dates
[ ] Mentioned these dates but did not mention Open Enrollment
[ ] Referred to wrong period with dates and/or provided wrong date range
[ ] No mention of Open Enrollment or these dates
[ ] N/A – I could not reach a counselor after two attempts

16. In response to "When can I select my Medicare coverage plan, and when can I change it later?", did the counselor mention the Annual Election Period (1/1-3/31)?
[ ] Yes, mentioned Annual Election Period and provided these dates
[ ] Mentioned Annual Election Period but did not provide these dates
[ ] Mentioned these dates but did mention Annual Election Period
[ ] Referred to wrong period with these dates and/or provided wrong date range
[ ] No mention of Annual Election Period or these dates
[ ] N/A – I could not reach a counselor after two attempts

17. In response to "When can I select my Medicare coverage plan, and when can I change it later?", did the counselor mention the Special Enrollment Period?
[ ] Yes
[ ] No
[ ] N/A – I could not reach a counselor after two attempts

18. Describe in detail the counselor's response when asked, "When can I select my Medicare coverage plan, and when can I change it later?"

19. Select the option that best describes the counselor's response when asked, "Do you offer Spanish translation services at your location?"
[ ] Yes
[ ] Yes, and listed other languages that are available
[ ] Yes, but only on certain days and times or by appointment
[ ] No, translation services are available, but not for Spanish
[ ] No, translation services are not available for any language
[ ] None of the above
[ ] N/A – I could not reach a counselor after two attempts

20. Describe in detail the counselor's response when asked, "Do you offer Spanish translation services at your location?"

Dual Eligible

21. Did the counselor mention original Medicare Part A and Part B when asked, "If I stay eligible for Medicaid, what are my options for enrolling in Medicare?"
[ ] Yes, both Medicare Part A and Part B
[ ] No, Medicare Part A but not Part B
[ ] No, Medicare Part B but not Part A
[ ] No, neither was mentioned
[ ] N/A – Medicare Eligible Only scenario or I could not reach a counselor after two attempts

22. Did the counselor mention that original Medicare benefits would be provided separately from your current Medicaid benefits?
[ ] Yes
[ ] No
[ ] N/A – original Medicare was not mentioned
[ ] N/A – Medicare Eligible Only scenario or I could not reach a counselor after two attempts

23. Did the counselor mention that with original Medicare, premiums, cost sharing, and deductibles would be covered by Medicaid, and you would not have to cover those costs yourself?
[ ] Yes
[ ] No
[ ] N/A – original Medicare was not mentioned

[ ] N/A – Medicare Eligible Only scenario or I could not reach a counselor after two attempts

24. What did the counselor say about original Medicare (Part A and Part B) when asked, "If I stay eligible for Medicaid, what are my options for enrolling in Medicare?"

25. Did the counselor mention optional prescription drug coverage (Part D) when asked, "If I stay eligible for Medicaid, what are my options for enrolling in Medicare?"

[ ] Yes, mentioned prescription drug coverage or Part D as optional
[ ] No, mentioned prescription drug coverage or Part D but did not specify 'optional'
[ ] No, did not mention prescription drug coverage or Part D
[ ] N/A – Medicare Eligible Only scenario or I could not reach a counselor after two attempts

26. Did the counselor mention that optional prescription drug coverage (Part D) premiums, cost sharing, and deductibles would be covered by Medicaid, and you would not have to cover those costs yourself?

[ ] Yes
[ ] No
[ ] N/A – prescription drug coverage (Part D) was not mentioned
[ ] N/A – Medicare Eligible Only scenario or I could not reach a counselor after two attempts

27. What did the counselor say about prescription drug coverage (Part D) when asked, "If I stay eligible for Medicaid, what are my options for enrolling in Medicare?"

28. Did the counselor mention Medicare Advantage Plans (Part C) when asked, "If I stay eligible for Medicaid, what are my options for enrolling in Medicare?"

[ ] Yes, mentioned Medicare Advantage (Part C)
[ ] No, did not mention Medicare Advantage (Part C)
[ ] N/A – Medicare Eligible Only scenario or I could not reach a counselor after two attempts

29. Did the counselor mention that with a Medicare Advantage Plan (Part C), your current Medicaid benefits would be provided separately?

[ ] Yes
[ ] No
[ ] N/A – Medicare Advantage (Part C) was not mentioned
[ ] N/A – Medicare Eligible Only scenario or I could not reach a counselor after two attempts

30. Did the counselor mention that with a Medicare Advantage Plan (Part C), premiums, cost sharing, and deductibles would be covered by Medicaid, and you would not have those costs?

[ ] Yes
[ ] No
[ ] N/A – Medicare Advantage (Part C) was not mentioned
[ ] N/A – Medicare Eligible Only scenario or I could not reach a counselor after two attempts

31. What did the counselor say about Medicare Advantage Plans (Part C) when asked, "If I stay eligible for Medicaid, what are my options for enrolling in Medicare?"

32. Did the counselor mention Dual Eligible Special Needs Plans (D-SNP) when asked, "If I stay eligible for Medicaid, what are my options for enrolling in Medicare?"

[ ] Yes
[ ] No
[ ] N/A – Medicare Eligible Only scenario or I could not reach a counselor after two attempts

33. Did the counselor mention that with a Dual Eligible Special Needs Plan (D-SNP), Medicare and most Medicaid benefits would be provided through one single plan?

[ ] Yes
[ ] No
[ ] N/A – Dual Eligible Special Needs Plans (D-SNP) were not mentioned
[ ] N/A – Medicare Eligible Only scenario or I could not reach a counselor after two attempts

34. Did the counselor mention that with a Dual Eligible Special Needs Plan (D-SNP), Medicare prescription drug coverage (Part D) would be included in that plan?

[ ] Yes
[ ] No
[ ] N/A – Dual Eligible Special Needs Plans (D-SNP) were not mentioned
[ ] N/A – Medicare Eligible Only scenario or I could not reach a counselor after two attempts

35. Did the counselor mention that with a Dual Eligible Special Needs Plan (D-SNP), there would be minimal or no premiums, cost

sharing, or deductibles?
[ ] Yes
[ ] No
[ ] N/A – Dual Eligible Special Needs Plans (D-SNP) were not mentioned
[ ] N/A – Medicare Eligible Only scenario or I could not reach a counselor after two attempts

36. What did the counselor say about Dual Eligible Special Needs Plans (D-SNP) when asked, "If I stay eligible for Medicaid, what are my options for enrolling in Medicare?"

37. If original Medicare, Medicare Advantage, prescription drug coverage, and special needs plans were not mentioned, describe the counselor's response when asked, "If I stay eligible for Medicaid, what are my options for enrolling in Medicare?"
Enter N/A if any of these were mentioned and you have no additional comments about the response you received to this question.

38. Which of the following did the counselor mention when asked, "I saw a commercial about special plans for people with both Medicaid and Medicare. Can you tell me more about them and why I may or may not want to choose one?"
Select all that apply.
[ ] Additional benefits
[ ] Better care coordination
[ ] Makes it easier to get most Medicare and Medicaid benefits through one plan
[ ] Dual eligible plans are specifically for dual eligible people
[ ] You may not be able to keep your doctor
[ ] You may have fewer options for doctors to choose from than traditional Medicare
[ ] You may have fewer options for prescription drugs you can get
[ ] None of the above
[ ] N/A – Medicare Eligible Only scenario or I could not reach a counselor after two attempts

39. How many advantages or disadvantages did the counselor mention when asked, "I saw a commercial about special plans for people with both Medicaid and Medicare. Can you tell me more about them and why I may or may not want to choose one?"
Select the option that matches the number of responses you selected in the previous question.
[ ] 0
[ ] 1
[ ] 2
[ ] 3
[ ] 4
[ ] 5
[ ] 6
[ ] 7
[ ] N/A – Medicare Eligible Only scenario or I could not reach a counselor after two attempts

40. Describe in detail the counselor's response when asked, "I saw a commercial about special plans for people with both Medicaid and Medicare. Can you tell me more about them and why I may or may not want to choose one?"

41. What was the counselor's response when asked, "Are special plans for people with Medicaid and Medicare available in my area?"
Your area = the city, state, zip code, and county listed in your shop confirmation
[ ] D-SNP available: Yes
[ ] D-SNP available: No
[ ] D-SNP unavailable: Yes
[ ] D-SNP unavailable: No
[ ] The counselor did not know and did not offer to confirm
[ ] None of the above
[ ] N/A – Medicare Eligible Only scenario or I could not reach a counselor after two attempts

42. If no plans were identified, describe the counselor's response when asked, "Are special plans for people with Medicaid and Medicare available in my area?"

43. Select the option that best describes the counselor's response when asked, "If I ever need long-term care in the future, would it be covered under any of the Medicare options we've talked about?"
[ ] Yes, long-term care would be covered under any of the options we've talked about, but only if you stay eligible for Medicaid and meet the criteria for long-term care
[ ] Yes, but did not clarify the requirement for Medicaid eligibility
[ ] No, Medicare does not cover long-term care, but Medicaid does, if you remain eligible
[ ] No, long-term care would not be covered under any of the options we've talked about
[ ] None of the above
[ ] N/A – Medicare Eligible Only scenario or I could not reach a counselor after two attempts
[ ] None of the above
[ ] N/A – Medicare Eligible Only scenario or I could not reach a counselor after two attempts

44. Which of the following did the counselor mention when asked, "If I ever need long-term care in the future, would it be covered under any of the Medicare options we've talked about?" Select all that apply.
[ ] Medicaid covers long-term care, but Medicare does not

[ ] Medicaid covers long-term care for people whose mental or physical condition requires nursing supervision/assistance with activities of daily living
[ ] Long-term care would be provided separately from whatever Medicare option you choose
[ ] Options for Medicare coverage that cover all Medicare and Medicaid benefits including fully integrated dually eligible special needs plans, or Programs for All-Inclusive Care for the Elderly
[ ] In general, Medicare does not cover long-term care, although some time-limited
[ ] None of the above
[ ] N/A – Medicare Eligible Only scenario or I could not reach a counselor after two attempts

45. Describe in detail the counselor's response when asked, "If I ever need long-term care in the future, would it be covered under any of the Medicare options we've talked about?"

46. Select the option that best describes the counselor's response when asked, "What about Medicaid? If I stay eligible, will Medicaid pay for long-term care?"
[ ] D-SNP available: Yes, if you meet the requirements for needing long-term care
[ ] D-SNP available: Yes (no further explanation)
[ ] D-SNP available: No
[ ] D-SNP unavailable: Yes, if you meet the requirements for needing long-term care
[ ] D-SNP unavailable: Yes (no further explanation)
[ ] D-SNP unavailable: No
[ ] The counselor did not know and did not offer to confirm
[ ] None of the above
[ ] N/A – Medicare Eligible Only scenario or I could not reach a counselor after two attempts

47. Describe in detail the counselor's response when asked, , "What about Medicaid? If I stay eligible, will Medicaid pay for long-term care?"

48. Select the counselor's response when asked, "If I stay eligible for Medicaid, will it pay for my Medicare premiums, cost sharing, and deductibles no matter if I choose a Dual Eligible Special Needs Plan, a Medicare Advantage Plan, or regular Medicare?"
[ ] Yes, if you are on Medicaid, you will have minimal or no premiums, cost sharing, or deductibles regardless of which Medicare option you choose
[ ] It depends on the plan chosen
[ ] None of the above
[ ] N/A – Medicare Eligible Only scenario or I could not reach a counselor after two attempts

49. Describe the response when asked, "If I stay eligible for Medicaid, will it pay for my Medicare premiums, cost sharing, and deductibles no matter if I choose a Dual Eligible Special Needs Plan, a Medicare Advantage Plan, or regular Medicare?"

50. Select the option that best describes the counselor's response when asked, "I was told that if I'm not eligible for Medicaid, there are other programs to help with out-of-pocket costs for Medicare. My income is $1,600 a month. Would I get any help?"
[ ] Discussed a specific program to help with Medicare Part B premiums, indicated you may be eligible for it, and noted that if you qualify, you would also qualify for a specific program to help with Medicare Part D prescription drug costs
[ ] Discussed a specific program to help with Medicare Part D prescription drug costs and indicated you may be eligible for it, but did not mention a program to help with Part B premiums
[ ] Discussed a specific program to help with Medicare Part B premiums and indicated you may be eligible for it, but did not mention a separate program to help with Medicare Part D prescription drug costs
[ ] Discussed one or more available programs to help with Medicare Part A or Part B premiums, deductibles, other cost sharing, and prescription drug (Part D) costs, but did not indicate whether (or for which ones) you would likely qualify
[ ] Provided high-level, non-specific information about other programs to help with some Medicare costs, but did not provide any further detail or information
[ ] Referred to an external source for an answer (e.g., state Medicaid office)
[ ] None of the above
[ ] N/A – Medicare Eligible Only scenario or I could not reach a counselor after two attempts

51. Describe in detail the counselor's response when asked, "I was told that if I'm not eligible for Medicaid, there are other programs to help with out-of-pocket costs for Medicare. My income is $1,600 a month. Would I get any help?"

Medicare Eligible Only

52. Select the option that best describes the counselor's response when asked, "Do I have to sign up for Medicare when I turn 65, or can I remain on my employer plan?"
[ ] No, you don't have to sign up for Medicare, but you may face a late enrollment penalty if you delay, depending on the size of your employer
[ ] No, you don't have to sign up for Medicare, but you may face a late enrollment penalty if you delay
[ ] No, you don't have to sign up for Medicare
[ ] Yes, you have to sign up for Medicare

[ ] None of the above
[ ] N/A – Dual Eligible scenario or I could not reach a counselor after two attempts

53. Did the counselor mention that if you enroll in Medicare, you can also remain covered by your employer plan?
[ ] Yes
[ ] No
[ ] N/A – Dual Eligible scenario or I could not reach a counselor after two attempts

54. Describe in detail the counselor's response when asked, "Do I have to sign up for Medicare when I turn 65, or can I remain on my employer plan?"

55. Which of the following did the counselor mention about Medicare Advantage Plans when asked, "How are Medicare Advantage Plans different from regular Medicare, and what are good and bad about them?"
Select all that apply.
[ ] May have lower premiums, deductibles, and cost sharing than traditional Medicare plus Supplement plan
[ ] Has a more restrictive choice of providers and hospitals
[ ] You may not be able to keep your doctor
[ ] Covers all Medicare Part A and Part B (sometimes Part D) benefits
[ ] One must continue to pay the Medicare Part B premium in addition to their Medicare Advantage Plan premium
[ ] Often offers additional benefits such as vision, hearing, dental, fitness, grocery, OTC card, etc.
[ ] None of the above
[ ] N/A – Dual Eligible scenario or I could not reach a counselor after two attempts

56. How many advantages or disadvantages did the counselor mention when asked, "How are Medicare Advantage Plans different from regular Medicare, and what are good and bad about them?"
Select the option that matches the number of responses you selected in the previous question.
[ ] 0
[ ] 1
[ ] 2
[ ] 3
[ ] 4
[ ] 5
[ ] 6
[ ] N/A – Dual Eligible scenario or I could not reach a counselor after two attempts

57. What did the counselor say about Medicare Advantage Plans (Part C) when asked, "How are Medicare Advantage Plans different from regular Medicare, and what are good and bad about them?"

58. Which of the following did the counselor mention about original Medicare when asked, "How are Medicare Advantage Plans different from regular Medicare, and what are good and bad about them?"
Select all that apply.
[ ] You automatically enroll in Medicare Part A when you apply for Medicare, which provides coverage for hospital stay
[ ] You have the option to enroll in Medicare Part B to cover doctors' services, outpatient care, and preventative services
[ ] Part A has a deductible and some cost sharing but no monthly premium
[ ] Part B has a monthly premium, some cost sharing, and a small deductible
[ ] Premiums and cost sharing can be more expensive than with Medicare Advantage Plans
[ ] Your choice of almost any provider, hospital, etc.
[ ] In order to receive coverage for prescription drugs, you must obtain a separate Prescription Drug Plan (Part D)
[ ] Can be paired with a Medigap/Medicare Supplement policy to reduce premiums, deductibles, and cost sharing
[ ] None of the above
[ ] N/A – Dual Eligible scenario or I could not reach a counselor after two attempts

59. How many advantages or disadvantages did the counselor mention when asked, "How are Medicare Advantage Plans different from regular Medicare, and what are good and bad about them?"
Select the option that matches the number of responses you selected in the previous question.
[ ] 0
[ ] 1
[ ] 2
[ ] 3
[ ] 4
[ ] 5
[ ] 6
[ ] 7
[ ] 8
[ ] N/A – Dual Eligible scenario or I could not reach a counselor after two attempts

60. What did the counselor say about original Medicare (Part A and Part B) when asked, "How are Medicare Advantage Plans different from regular Medicare, and what are good and bad about them?"

61. If Medicare Advantage and original Medicare were not discussed, describe the counselor's response when asked, "How are Medicare Advantage Plans different from regular Medicare, and what are good and bad about them?"
Enter N/A if either of these was discussed and you have no additional comments about the response you received to this question.

62. Which of the following did the counselor mention about Medicare Supplement Plans when asked, "What is a Medicare Supplement Plan, how much do they cost, and when do I enroll?"

Select all that apply.

[ ] Supplement plans can be purchased separately if you choose traditional Medicare to help cover out-of-pocket costs
[ ] All supplement plans cover Part A and Part B copays and hospital and hospice costs
[ ] Some supplement plans cover Part A and Part B deductibles
[ ] Some supplement plans have out-of-pocket limits
[ ] Supplement plan costs vary based on how much is covered but usually range from $50-$300 per month
[ ] You can enroll in a Supplement plan at any time
[ ] You will get better rates if you enroll during your open enrollment period (which begins the day you turn 65 and lasts for six months afterwards)
[ ] None of the above
[ ] N/A – Dual Eligible scenario or I could not reach a counselor after two attempts

63. How many topics did the counselor mention when asked, "What is a Medicare Supplement Plan, how much do they cost, and when do I enroll?"

Select the option that matches the number of responses you selected in the previous question.

[ ] 0
[ ] 1
[ ] 2
[ ] 3
[ ] 4
[ ] 5
[ ] 6
[ ] 7
[ ] N/A – Dual Eligible scenario or I could not reach a counselor after two attempts

64. Describe in detail the counselor's response when asked, "What is a Medicare Supplement Plan, how much do they cost, and when do I enroll?"

65. Select the option that best describes the counselor's response when asked, "Do either original Medicare, Medicare Advantage Plans, or Medicare Supplement Plans cover long-term care?"

[ ] In general, none of these cover long-term care, although some time-limited nursing facility stays or in-home services may be covered
[ ] No, none of these provide coverage for long-term care
1[ ] Yes, under one or more of these, long-term care is covered
[ ] None of the above
[ ] N/A – Dual Eligible scenario or I could not reach a counselor after two attempts

66. Describe in detail the counselor's response when asked, "Do either original Medicare, Medicare Advantage Plans, or Medicare Supplement Plans cover long-term care?"

67. Which of the following considerations did the counselor mention when asked, "What are my options for Medicare prescription drug coverage, and how should I choose?"

Select all that apply.

[ ] You can choose either a Medicare Advantage Plan that covers prescription drugs or a stand-alone Part D plan
[ ] Most Medicare Advantage options include prescription drug (Part D) coverage
[ ] If you choose original Medicare, you could purchase a separate prescription drug (Part D) plan
[ ] Each plan has a different list of covered drugs, and you would choose based on the medicine you take
[ ] Each plan has different out-of-pocket costs for different drugs, and you would choose based on the medicine you take and your comfort with costs
[ ] None of the above
[ ] N/A – Dual Eligible scenario or I could not reach a counselor after two attempts

68. How many considerations did the counselor mention when asked, "What are my options for Medicare prescription drug coverage, and how should I choose?"

Select the option that matches the number of responses you selected in the previous question.

[ ] 0
[ ] 1
[ ] 2
[ ] 3
[ ] 4
[ ] 5
[ ] N/A – Dual Eligible scenario or I could not reach a counselor after two attempts

69. Which of the following prescription drug coverage (or Part D) topics were mentioned?

Select all that apply.

[ ] Late enrollment penalty
[ ] Low income subsidy (known as LIS or 'extra help')
[ ] Drug tiers
[ ] Formulary
[ ] Step therapy
[ ] Prior authorization
[ ] Copayments
[ ] Brand name vs. generic drugs
[ ] Prescription drug stages (initial coverage, coverage gap/donut hole, and/or catastrophic coverage)

[ ] Exceptions (what to do if your drug isn't listed in the formulary)
[ ] None of the above
[ ] N/A – Dual Eligible scenario or I could not reach a counselor after two attempts

70. How many prescription drug coverage (Part D) topics did the counselor mention?
Select the option that matches the number of responses you selected in the previous question.
[ ] 0
[ ] 1
[ ] 2
[ ] 3
[ ] 4
[ ] 5
[ ] 6
[ ] 7
[ ] 8
[ ] 9
[ ] 10
[ ] N/A – Dual Eligible scenario or I could not reach a counselor after two attempts

71. Describe in detail the counselor's response when asked, "What are my options for Medicare prescription drug coverage, and how should I choose?"

72. What was the counselor's response when asked, "My friend told me about [plan name]. Can you tell me if [doctor name] is in the network there?"
[ ] The doctor is in the network
[ ] The doctor is not in the network
[ ] Neither of the above
[ ] N/A – Dual Eligible scenario or I could not reach a counselor after two attempts

73. Describe in detail the counselor's response when asked, "My friend told me about [plan name]. Can you tell me if [doctor name] is in the network there?"

74. Select the option that best describes the counselor's response when asked, "What is the monthly premium for [plan name]?
[ ] Provided the plan premium cost and the Part B premium cost separately
[ ] Provided one total combined cost (plan premium plus Part B premium)
[ ] Provided only the plan premium (Part B premium not provided)
[ ] Provided only the Part B premium (plan premium not provided)
[ ] None of the above
[ ] Provided the wrong dollar amount
[ ] N/A – Dual Eligible scenario or I could not reach a counselor after two attempts

75. What dollar amount(s) did the counselor provide for the monthly premium, or if the monthly premium was not provided, what did the counselor say when asked, "What is the monthly premium for [plan name]?

76. What was the counselor's response when asked, "Would [plan name] let me go out of network?"
[ ] PPO: Yes, but with higher cost sharing
[ ] PPO: Yes, with no mention of higher cost sharing
[ ] PPO: No, plan would not allow me to go out of network
[ ] HMO: No, plan would not allow me to go out of network
[ ] HMO: Yes, but with higher cost sharing
[ ] HMO: Yes, with no mention of higher cost sharing
[ ] None of the above
[ ] N/A – Dual Eligible scenario or I could not reach a counselor after two attempts

77. Describe in detail the counselor's response when asked, "Would [plan name] let me go out of network?"

78. Did the counselor provide the <u>in-network</u> copay dollar amount when asked, "What is the copay for a primary care doctor visit for [plan name]?"
[ ] Yes, provided a specific dollar amount
[ ] Yes, provided a dollar range
[ ] No, in-network copay dollar amount not provided
[ ] Provided the wrong dollar amount
[ ] N/A – Dual Eligible scenario or I could not reach a counselor after two attempts

79. What was the copay amount given for an <u>in-network</u> primary care doctor visit, or what was said about in-network primary care doctor visits if no amount was given?

80. Did the counselor provide the <u>out-of-network</u> copay dollar amount when asked, "What is the copay for a primary care doctor visit for [plan name]?
[ ] PPO: Yes, provided a specific dollar amount
[ ] PPO: Yes, provided a dollar range
[ ] PPO: No, out-of-network copay dollar amount not provided
[ ] HMO: The counselor did not mention or provide an out-of-network copay amount
[ ] HMO: The counselor said the plan does not cover out-of-network visits
[ ] HMO: Yes, provided a specific dollar amount
[ ] HMO: Yes, provided a dollar range
[ ] Provided the wrong dollar amount

[ ] N/A – Dual Eligible scenario or I could not reach a counselor after two attempts

81. What was the copay amount given for an out-of-network primary care doctor visit, or what was said about out-of-network primary care doctor visits if no amount was given?

82. Did the counselor provide the in-network copay dollar amount when asked, "What is the copay to see a specialist for [plan name]?

[ ] Yes, provided a specific dollar amount
[ ] Yes, provided a dollar range
[ ] No, in-network copay dollar amount not provided
[ ] Provided the wrong dollar amount
[ ] N/A – Dual Eligible scenario or I could not reach a counselor after two attempts

83. What was the copay amount given for an in-network specialist visit, or what was said about in-network specialist visits if no amount was given?

84. Did the counselor provide the out-of-network copay dollar amount when asked, "What is the copay to see a specialist for [plan name]?

[ ] PPO: Yes, provided a specific dollar amount
[ ] PPO: Yes, provided a dollar range
[ ] PPO: No, out-of-network copay dollar amount not provided
[ ] HMO: The counselor did not mention or provide an out-of-network copay amount
[ ] HMO: The counselor said the plan does not cover out-of-network visits
[ ] HMO: Yes, provided a specific dollar amount
[ ] HMO: Yes, provided a dollar range
[ ] None of the above
[ ] Provided the wrong dollar amount
[ ] N/A – Dual Eligible scenario or I could not reach a counselor after two attempts

85. What was the copay amount given for an out-of-network specialist visit, or what was said if about out-of-network specialist visits if no amount was given?

86. Which of the following best describes the counselor's response when asked, "For [plan name], is there a limit on out-of-pocket costs I would have to pay each year?"

[ ] PPO: The counselor provided two separate dollar amounts: one for in-network care and one for out-of-network care and specified that certain costs do not count toward the limits, including amounts you pay in premiums and for prescription drugs
[ ] PPO: The counselor provided two separate dollar amounts: one for in-network care and one for out-of-network care, but did not specify that certain costs do not count toward the limits
[ ] PPO: The counselor provided a dollar amount but did not differentiate between in-network or out-of-network
[ ] PPO: The counselor indicated there is a maximum, but did not provide a dollar amount
[ ] PPO: The counselor said there is no maximum
[ ] HMO: The counselor provided a dollar amount and specified that certain costs do not count toward the limit, including amounts you pay in premiums, for prescription drugs, or for out-of-network care
[ ] HMO: The counselor provided a dollar amount, but did not specify which costs do not count toward the limit
[ ] HMO: The counselor indicated there is a maximum, but did not provide a dollar amount
[ ] HMO: The counselor said there is no maximum
[ ] None of the above
[ ] Provided the wrong dollar amount
[ ] N/A – Dual Eligible scenario or I could not reach a counselor after two attempts

87. What dollar amount(s) did the counselor provide for the maximum out-of-pocket costs, or if no dollar amount was given, what did the counselor say when asked, "For [plan name], is there a limit on out-of-pocket costs I would have to pay each year?"

88. What was the counselor's response when asked, "Does [plan name] include prescription drug coverage?"

[ ] The plan includes prescription drug coverage
[ ] The plan does not include prescription drug coverage
[ ] Neither of the above
[ ] N/A – Dual Eligible scenario or I could not reach a counselor after two attempts

89. Describe in detail the counselor's response when asked, "Does [plan name] include prescription drug coverage?"

90. Select the option that best describes the counselor's response when asked, "I take Lipitor. Is that covered by [plan name]?"

[ ] No, Lipitor is not covered, but the generic version is covered
[ ] No, Lipitor is not covered, and there was no mention of the generic version
[ ] Yes, Lipitor is covered
[ ] None of the above
[ ] N/A – Dual Eligible scenario or I could not reach a counselor after two attempts

91. If the generic version of Lipitor was not mentioned, what did the counselor say when asked if a generic version of Lipitor is covered?

[ ] Yes, a generic version is covered
[ ] No, a generic version is not covered
[ ] Neither of the above
[ ] N/A – a generic version was mentioned
[ ] N/A – Dual Eligible scenario or I could not reach a counselor after two attempts

92. Describe in detail the counselor's response when asked, "I take Lipitor. Is that covered by [plan name]?"

93. Were the costs the counselor provided for Lipitor and the generic version correct?
Check your shop confirmation to find the correct costs.

[ ] Yes, the costs for Lipitor and the generic version were both correct
[ ] The cost for Lipitor was not correct
[ ] The cost for the generic version was not correct
[ ] No, neither the cost for Lipitor nor the cost for the generic version was correct
[ ] N/A – costs not provided
[ ] N/A – Dual Eligible scenario or I could not reach a counselor after two attempts

94. What did the counselor say was the cost of Lipitor vs. the generic version, or if costs were not provided, what did the counselor say when asked, "How much would Lipitor be out of pocket vs. the generic version?"

General Information

95. Which of the following best describes how the counselor responded to your questions?

[ ] Full awareness (spoke confidently/knowledgeably – answered all questions)
[ ] Limited awareness but helpful (spoke with hesitation or uncertainty but found answers to questions not known)
[ ] Limited awareness and not helpful (spoke with hesitation or uncertainty and did not find answers to unknown questions)
[ ] No awareness but helpful (could not answer questions but provided a resource)
[ ] No awareness and not helpful (could not answer questions and did not provide a resource)
[ ] N/A – I could not reach a counselor after two attempts

96. Please explain your response:

97. If the counselor directed you to another resource, which were you directed to?
Select all that apply.

[ ] State Medicaid office
[ ] State toll-free line
[ ] Medicare.gov
[ ] Plan representative
[ ] Other
[ ] I was not directed to another resource
[ ] N/A – I could not reach a counselor after two attempts

98. Please explain what was said when you were directed to another resource:

99. If you could not reach a counselor after two attempts, explain all your attempts to contact a counselor and any other details you have about why you could not complete the shop:

100. Location classification:
Do not answer. CX Group will populate.

[ ] Low income, small number of plans
[ ] Low income, large number of plans
[ ] High income, small number of plans
[ ] High income, large number of plans

101. Health plan name:
Enter the health plan name listed in your shop confirmation.
Enter N/A if you completed the Dual Eligible scenario.

102.Health plan ID number:
Do not answer. CX Group will populate.

103.Enter the city you provided to the counselor:
If you did not provide a city, enter the city found in your shop confirmation.

104. Enter the state you provided to the counselor:
If you did not provide a state, enter the state found in your shop confirmation.

105. Enter the zip code you provided to the counselor:
If you did not provide a zip code, enter the zip code found in your shop confirmation.

106. Enter the county you provided to the counselor:
If you did not provide a county, enter the county found in your shop confirmation.

107. What time zone did you make the call from?
] Eastern Time Zone
[ ] Central Time Zone
[ ] Mountain Time Zone
[ ] Pacific Time Zone
[ ] Hawaiian Time Zone
[ ] Alaskan Time Zone

108. IVR confirmation number(s):

END OF QUESTIONNAIRE

eAppendix4. Scoring Guide

The scoring guide below was used to assign one of six scores to a counselor's answer on each of 20 question groups:

To assess accuracy and completeness, we developed a scoring system that used close-ended questionnaire data to categorize counselor responses to a series of 20 unique question groups into one of six categories:

1. Accurate and Complete
2. Substantive but Incomplete
3. Not Substantive
4. Incorrect
5. Needs Review
6. Missing/incomplete

We used Stata 16 IC to score the accuracy and completion of information provided by counselors.

The category "Needs Review" was used to identify instances where counselor responses did not fully align with the structure of the questionnaire and scoring system. For answers scored as "5. Needs review," we implemented a two-layer review process in which two analysts independently reviewed complete shop data to determine how responses should be classified. Disagreements in classification were resolved through discussion until consensus was reached.

We coded an answer as incorrect only if an incorrect detail materially changed the information provided to the shopper or would materially change how a Medicare enrollee might make decisions.

1. Not scored
2. Not scored
3. Not scored
4. Not scored
5. Not scored
6. Not scored
7. Not scored
8. Not scored
9. Not scored
10. Not scored
11. Not scored
12. Not scored
13. Not scored

Both Scenarios

<u>Question Group 1: Questions 14 – 16, 18</u>

Script Questions/Topics

Medicare-Only Question #1 When can I select my Medicare coverage plan, and when can I change it later?

- Topic (Medicare): Timing for Initial Medicare Enrollment & Subsequent Changes

Dual-Eligible Question #1 When can I select my Medicare coverage plan, and when can I change it later?

- Topic (Dual eligible): Timing for Initial Medicare Enrollment & Subsequent Changes

14. In response to "When can I select my Medicare coverage plan, and when can I change it later?", did the counselor mention you can select Medicare coverage within 3 months of turning 65?
    1. Yes
    2. No
    3. N/A – I could not reach a counselor after two attempts

15. In response to "When can I select my Medicare coverage plan, and when can I change it later?", did the counselor mention the Open Enrollment Period (10/15-12/7)?
    1. Yes, mentioned Open Enrollment and provided these dates
    2. Mentioned Open Enrollment but did not provide these dates
    3. Mentioned these dates but did not mention Open Enrollment
    4. Referred to wrong period with dates and/or provided wrong date range
    5. No mention of Open Enrollment or these dates
    6. N/A – I could not reach a counselor after two attempts
16. In response to "When can I select my Medicare coverage plan, and when can I change it later?", did the counselor mention the Annual Election Period (1/1-3/31)?
    1. Yes, mentioned Annual Election Period and provided these dates
    2. Mentioned Annual Election Period but did not provide these dates
    3. Mentioned these dates but did mention Annual Election Period
    4. Referred to wrong period with these dates and/or provided wrong date range
    5. No mention of Annual Election Period or these dates
    6. N/A – I could not reach a counselor after two attempts
17. Not scored
18. Describe in detail the counselor's response when asked, "When can I select my Medicare coverage plan, and when can I change it later?"

Scoring Guide

1. Accurate and Complete if:
    a. Question 14: 1
    b. AND
    c. Question 15: 1, 2, or 3 OR Question 16: 1, 2, or 3
2. Substantive but Incomplete
    a. Question 14: 1
    b. AND
    c. Question 15: 5, OR Question 16: 5
3. Not Substantive
    a. Question 14: 2
    b. AND
    c. Question 15: 5 AND Question 16: 5
4. Incorrect
5. Needs Review
    a. Review of Question 18 needed if any other combination of answer options is selected.
    b. Reviewer instructions:
        i. Score as "accurate and complete" if the counselor's response:
            1. Mentioned that you can enroll in Medicare/select coverage within 3 months of turning 65
            2. Substantively answered the "when can I change it" question by referencing open enrollment period, annual election period, or special enrollment periods.
        ii. Score as "substantive but incomplete" if some substantive information was provided but both components of the question were not answered (e.g., saying shopper can enroll "now" because counselors were instructed to provide a date of birth within three months of their call, but not addressing the question of when a shopper can make changes).
        iii. Score as "not substantive" if the counselor did not answer the question (e.g., said they did not know, or declined to answer without shopper's Medicare card).
        iv. Score as incorrect if incorrect information was provided, and the incorrect information was substantive enough to materially change the substance of the counselor's answer or affect an enrollee's decision.
        v. Score as Missing/N/A if shopper could not ask the question

<u>Question Group 2: Questions 19 – 20</u>

Script Questions/Topics

Medicare-Only Question #16: I have a friend who does not speak much English. Do you offer Spanish translation services at your location?

- Topic (Medicare-Only): Availability of Spanish Translation Services

Dual-Eligible Question #8: I have a friend who does not speak much English. Do you offer Spanish translation services at your location?

- Topic (Dual-Eligible): Availability of Spanish Translation Services

19. Select the option that best describes the counselor's response when asked, "Do you offer Spanish translation services at your location?"
    1. Yes
    2. Yes, and listed other languages that are available
    3. Yes, but only on certain days and times or by appointment
    4. No, translation services are available, but not for Spanish
    5. No, translation services are not available for any language
    6. None of the above
    7. N/A – I could not reach a counselor after two attempts
20. Describe in detail the counselor's response when asked, "Do you offer Spanish translation services at your location?"

Scoring Guide

1. Yes if:
    a. Question 19: 1, 2, or 3
2. No if
    a. Question 19: 4 or 5
3. Needs Review
    a. Question 19: 6
    b. Reviewer instructions:
        i. Score as "Yes" if counselor substantively said yes
        ii. Score as "No" if counselor said no, even if they offered a workaround or a way to provide some degree of translation services (e.g., offering to bring in a friend to translate).
        iii. Score as "Unsure" if counselor did not know
        iv. Score Missing/N/A if shopper could not ask the question

Dual-Eligible Scenario

<u>Question Group 3: Questions 21-37</u>

Script Questions/Topics

Dual-Eligible Question #2: If I stay eligible for Medicaid, what are my options for enrolling in Medicare?

- Topic: Options for Enrolling in Medicare if Remaining Eligible for Full Medicaid Benefits

21. Did the counselor mention original Medicare Part A and Part B when asked, "If I stay eligible for Medicaid, what are my options for enrolling in Medicare?"
    1. Yes, both Medicare Part A and Part B
    2. No, Medicare Part A but not Part B
    3. No, Medicare Part B but not Part A
    4. No, neither was mentioned
    5. N/A – Medicare Eligible Only scenario or I could not reach a counselor after two attempts

22. Did the counselor mention that original Medicare benefits would be provided separately from your current Medicaid benefits?
    1. Yes
    2. No
    3. N/A – original Medicare was not mentioned
    4. N/A – Medicare Eligible Only scenario or I could not reach a counselor after two attempts
23. Did the counselor mention that with original Medicare, premiums, cost sharing, and deductibles would be covered by Medicaid, and you would not have to cover those costs yourself?
    1. Yes
    2. No
    3. N/A – original Medicare was not mentioned
    4. N/A – Medicare Eligible Only scenario or I could not reach a counselor after two attempts
24. What did the counselor say about original Medicare (Part A and Part B) when asked, "If I stay eligible for Medicaid, what are my options for enrolling in Medicare?"

25. Did the counselor mention optional prescription drug coverage (Part D) when asked, "If I stay eligible for Medicaid, what are my options for enrolling in Medicare?"
    1. Yes, mentioned prescription drug coverage or Part D as optional
    2. No, mentioned prescription drug coverage or Part D but did not specify 'optional'
    3. No, did not mention prescription drug coverage or Part D
    4. N/A – Medicare Eligible Only scenario or I could not reach a counselor after two attempts
26. Did the counselor mention that optional prescription drug coverage (Part D) premiums, cost sharing, and deductibles would be covered by Medicaid, and you would not have to cover those costs yourself?
    1. Yes
    2. No
    3. N/A – prescription drug coverage (Part D) was not mentioned
    4. N/A – Medicare Eligible Only scenario or I could not reach a counselor after two attempts
27. What did the counselor say about prescription drug coverage (Part D) when asked, "If I stay eligible for Medicaid, what are my options for enrolling in Medicare?"

28. Did the counselor mention Medicare Advantage Plans (Part C) when asked, "If I stay eligible for Medicaid, what are my options for enrolling in Medicare?"
    1. Yes, mentioned Medicare Advantage (Part C)
    2. No, did not mention Medicare Advantage (Part C)
    3. N/A – Medicare Eligible Only scenario or I could not reach a counselor after two attempts

29. Did the counselor mention that with a Medicare Advantage Plan (Part C), your current Medicaid benefits would be provided separately?
    1. Yes
    2. No
    3. N/A – Medicare Advantage (Part C) was not mentioned
    4. N/A – Medicare Eligible Only scenario or I could not reach a counselor after two attempts

30. Did the counselor mention that with a Medicare Advantage Plan (Part C), premiums, cost sharing, and deductibles would be covered by Medicaid, and you would not have those costs?
    1. Yes
    2. No
    3. N/A – Medicare Advantage (Part C) was not mentioned
    4. N/A – Medicare Eligible Only scenario or I could not reach a counselor after two attempts

31. What did the counselor say about Medicare Advantage Plans (Part C) when asked, "If I stay eligible for Medicaid, what are my options for enrolling in Medicare?"

32. Did the counselor mention Dual Eligible Special Needs Plans (D-SNP) when asked, "If I stay eligible for Medicaid, what are my options for enrolling in Medicare?"
    1. Yes

2. No
3. N/A – Medicare Eligible Only scenario or I could not reach a counselor after two attempts

33. Did the counselor mention that with a Dual Eligible Special Needs Plan (D-SNP), Medicare and most Medicaid benefits would be provided through one single plan?
    1. Yes
    2. No
    3. N/A – Dual Eligible Special Needs Plans (D-SNP) were not mentioned
    4. N/A – Medicare Eligible Only scenario or I could not reach a counselor after two attempts

34. Did the counselor mention that with a Dual Eligible Special Needs Plan (D-SNP), Medicare prescription drug coverage (Part D) would be included in that plan?
    1. Yes
    2. No
    3. N/A – Dual Eligible Special Needs Plans (D-SNP) were not mentioned
    4. N/A – Medicare Eligible Only scenario or I could not reach a counselor after two attempts

35. Did the counselor mention that with a Dual Eligible Special Needs Plan (D-SNP), there would be minimal or no premiums, cost sharing, or deductibles?
    1. Yes
    2. No
    3. N/A – Dual Eligible Special Needs Plans (D-SNP) were not mentioned
    4. N/A – Medicare Eligible Only scenario or I could not reach a counselor after two attempts

36. What did the counselor say about Dual Eligible Special Needs Plans (D-SNP) when asked, "If I stay eligible for Medicaid, what are my options for enrolling in Medicare?"

37. If original Medicare, Medicare Advantage, prescription drug coverage, and special needs plans were not mentioned, describe the counselor's response when asked, "If I stay eligible for Medicaid, what are my options for enrolling in Medicare?"

Scoring Guide

1. Accurate and Complete if:
    a. Question 21: 1
    b. AND
    c. Question 22: 1
    d. AND
    e. Question 23: 1
    f. AND
    g. Question 25: 1 or 2
    h. AND
    i. Question 26: 1
    j. AND
    k. Question 28: 1
    l. AND
    m. Question 29: 1
    n. AND
    o. Question 30: 1
    p. AND
    q. Question 32: 1
    r. AND
    s. Question 33: 1
    t. AND
    u. Question 34: 1
    v. AND

    w. Question 35: 1
2. Substantive but Incomplete
3. Not Substantive
    a. Question 21: 4
    b. AND
    c. Question 22: 2 or 3
    d. AND
    e. Question 23: 2 or 3
    f. AND
    g. Question 25: 3
    h. AND
    i. Question 26: 2 or 3
    j. AND
    k. Question 28: 2
    l. AND
    m. Question 29: 2 or 3
    n. AND
    o. Question 30: 2 or 3
    p. AND
    q. Question 32: 2
    r. AND
    s. Question 33: 2 or 3
    t. AND
    u. Question 34: 2 or 3
    v. AND
    w. Question 35: 2 or 3
4. Incorrect
5. Needs Review
    a. Review of Questions 24, 27, 31, 36, and 37 needed if any other combination of answer options is selected.
    b. Reviewer instructions:
        i. Score as accurate and complete if all of the topics mentioned in multiple choice questions 21 – 35 were substantively covered in the counselor's response, even if they did not use the same wording.
        ii. Score as substantive but incomplete if any (but not all) of the topics mentioned in multiple choice questions 21 – 35 were substantively covered in the counselor's response, even if they did not use the same wording.
        iii. Score as not substantive if the counselor did not discuss any of the topics mentioned in multiple choice questions 21 – 35 or did not answer the question.
        iv. Score as incorrect if incorrect information was provided, and the incorrect information was substantive enough to materially change the substance of the counselor's answer or affect an enrollee's coverage decision.
        v. Score Missing/N/A if shopper could not ask the question

<u>Question Group 4: Questions 38 & 40</u>

Script Questions/Topics

Dual-Eligible Question #3: I saw a commercial about special plans for people with both Medicaid and Medicare. Can you tell me more about them and why I may or may not want to choose one?

- Topic: Considerations for Selecting a D-SNP

38. Which of the following did the counselor mention when asked, "I saw a commercial about special plans for people with both Medicaid and Medicare. Can you tell me more about them and why I may or may not want to choose one?"

1. Additional benefits
2. Better care coordination
3. Makes it easier to get most Medicare and Medicaid benefits through one plan
4. Dual eligible plans are specifically for dual eligible people
5. You may not be able to keep your doctor
6. You may have fewer options for doctors to choose from than traditional Medicare
7. You may have fewer options for prescription drugs you can get
8. None of the above
9. N/A – Medicare Eligible Only scenario or I could not reach a counselor after two attempts

39. Not scored
40. Describe in detail the counselor's response when asked, "I saw a commercial about special plans for people with both Medicaid and Medicare. Can you tell me more about them and why I may or may not want to choose one?"

Scoring Guide

1. Accurate and Complete if:
    a. Question 38: 1 and 2 and 3 and 4 and 5 and 6 and 7
2. Substantive but Incomplete
    a. Question 38: 1 or 2 or 3 or 4 or 5 or 6 or 7
3. Not Substantive
4. Incorrect
5. Needs Review
    a. Review of Question 40 if Question 38: 8
    b. Reviewer instructions:
        i. Score as accurate and complete if all of the topics mentioned in multiple choice question 38 were substantively covered in the counselor's response, even if they did not use the same wording.
        ii. Score as substantive but incomplete if any (but not all) of the topics mentioned in multiple choice question 38 were substantively covered in the counselor's response, even if they did not use the same wording.
        iii. Score as not substantive if the counselor did not discuss any of the topics mentioned in multiple choice question 38 or did not answer the question (including if they speculated about Medicaid eligibility without offering any additional information to answer the question, or directed the shopper to Medicare.gov or Medicare hotline without offering additional information to answer the question).
        vi. Score as incorrect if incorrect information was provided, and the incorrect information was substantive enough to materially change the substance of the counselor's answer or affect an enrollee's coverage decision.
        vii. Score Missing/N/A if shopper could not ask the question

<u>**Question Group 5: Questions 41 & 42**</u>

Script Questions/Topics

Dual-Eligible Question #4: Are special plans for people with Medicaid and Medicare available in my area?

- Topic: D-SNP Availability in Shopper's Area

41. What was the counselor's response when asked, "Are special plans for people with Medicaid and Medicare available in my area?"

1. D-SNP available: Yes
2. D-SNP available: No
3. D-SNP unavailable: Yes
4. D-SNP unavailable: No
5. The counselor did not know and did not offer to confirm

6. None of the above
7. N/A – Medicare Eligible Only scenario or I could not reach a counselor after two attempts

42. If no plans were identified, describe the counselor's response when asked, "Are special plans for people with Medicaid and Medicare available in my area?"

Scoring Guide

1. Accurate and Complete if:
    a. Question 41: 1
2. Substantive but Incomplete
3. Not Substantive
    a. Question 41: 5
4. Incorrect
5. Needs Review
    a. Review of current D-SNP availability in Zip Code if Question 41: 2 or 3 or 4
    b. Review of Question 42 if Question 41: 6
    c. Reviewer instructions:
        i. Score as accurate and complete if the counselor's response substantively answered the question and their answer on D-SNP availability matches the results of Medicare.gov plan finder tool using the zip code of the SHIP site.
        ii. Score as substantive but incomplete if D-SNPs are not available in the zip code of the SHIP site, and the counselor provided information about Medicare Savings Programs/Extra Help rather than D-SNPs without noting that D-SNPs are not available.
        iv. Score as not substantive if the counselor did not substantively answer the question (including if they speculated about Medicaid eligibility without offering any additional information to answer the question, or directed the shopper to Medicare.gov or Medicare hotline without offering additional information to answer the question).
        viii. Score as incorrect if incorrect information was provided and the incorrect information was substantive enough to materially change the substance of the counselor's answer or affect an enrollee's coverage decision (including if D-SNP availability in the SHIP site's zip code contrasted with availability shown in the Medicare.gov plan finder tool using the zip code of the SHIP site).
        ix. Score Missing/N/A if shopper could not ask the question

43. Not scored
44. Not scored

<u>Question Group 6: Questions 45 & 47</u>

Script Questions/Topics

Dual-Eligible Question #5a: If I ever need long-term care in the future, would it be covered under any of the Medicare options we've talked about?

Follow-up question #5b: What about Medicaid? If I stay eligible, will Medicaid pay for long-term care?

- Topic: Coverage for Long-Term Care

45. Describe in detail the counselor's response when asked, "If I ever need long-term care in the future, would it be covered under any of the Medicare options we've talked about?"
46. Not scored
47. Describe in detail the counselor's response when asked, "What about Medicaid? If I stay eligible, will Medicaid pay for long-term care?"

Scoring Guide

1. Accurate and Complete if:
2. Substantive but Incomplete
3. Not Substantive
4. Incorrect
5. Needs Review
    a. Review of Questions 45 & 47 needed
    b. Reviewer instructions:
        i. Score as accurate and complete if across narrative answers for questions 45 and 47, the counselor substantively communicates that Medicare does not cover long-term care, but Medicaid does if one meets the financial criteria *and* criteria for medical/functional need.
        ii. Score as substantive but incomplete if counselor provides some substantive and accurate information, but not all information needed for accurate and complete (for example, if the counselor says that Medicare does not cover long-term care but Medicaid does, without specifying that one must meet additional financial and medical/functional criteria; if the counselor says simply that Medicare does not cover long-term care; if the counselor says that Medicare only covers time-limited nursing facility stays in certain circumstances, etc.)
        iii. Score as not substantive if the counselor says they do not know or otherwise does not substantively answer the question.
        iv. Score as incorrect if incorrect information was provided, and the incorrect information was substantive enough to materially change the substance of the counselor's answer or affect an enrollee's coverage decision. This includes if the counselor says that neither covers long-term care and coverage for long-term care requires separate insurance, or says or implies that Medicare does cover long-term care without clarifying the limited circumstances in which it does cover in-home or nursing facility services.
        v. Score Missing/N/A if shopper could not ask the question

<u>Question Grouping 7: Questions 48 & 49.</u>

Script Questions/Topics

Dual-Eligible Question #6: If I stay eligible for Medicaid, will it pay for my Medicare premiums and cost sharing and deductibles no matter if I choose a Dual Eligible Special Needs Plan, a Medicare Advantage Plan, or regular Medicare?

- Topic: Medicaid Coverage of Medicare Premiums and Cost Sharing

48. Select the counselor's response when asked, "If I stay eligible for Medicaid, will it pay for my Medicare premiums, cost sharing, and deductibles no matter if I choose a Dual Eligible Special Needs Plan, a Medicare Advantage Plan, or regular Medicare?"
    1. Yes, if you are on Medicaid, you will have minimal or no premiums, cost sharing, or deductibles regardless of which Medicare option you choose
    2. It depends on the plan chosen
    3. None of the above
    4. N/A – Medicare Eligible Only scenario or I could not reach a counselor after two attempts
49. Describe the response when asked, "If I stay eligible for Medicaid, will it pay for my Medicare premiums, cost sharing, and c no matter if I choose a Dual Eligible Special Needs Plan, a Medicare Advantage Plan, or regular Medicare?"
50. Not scored

Scoring Guide

1. Accurate and Complete if:
    a. Question 48: 1

2. Substantive but Incomplete
3. Not Substantive
4. Incorrect
5. Needs Review
    a. Review of Question 49 needed if Question 48: 2 or 3
    b. Reviewer instructions:
        i. Score as accurate and complete if the counselor substantively says the same thing as answer 1 in multiple choice question 48, indicating that if the shopper is eligible for full Medicaid benefits, they would not have to pay premiums, cost sharing, or deductibles (even if they do not use the same wording as in answer 1).
        ii. Score as substantive but incomplete if some (but not all) accurate information is provided; for example, if the counselor says that it "depends on the plan" or some variation and elaborates about what type of Medicaid program the shopper might qualify for such as Qualified Medicare Beneficiary; or if the counselor says that Medicaid would cover Part A premiums but cannot speak to other elements of Medicare cost sharing, or discuss Medicare Advantage implications.
        iii. Score as not substantive if counselor does not substantively answer the question, including if they say "it depends on the plan chosen" without elaborating further.
        iv. Score as incorrect if incorrect information was provided, and the incorrect information was substantive enough to materially change the substance of the counselor's answer or affect an enrollee's coverage decision.
        v. Score Missing/N/A if shopper could not ask the question.

<u>Question Grouping 8: Question 51</u>

Script Questions/Topics

Dual-Eligible Question #7: I was told that if I'm not eligible for Medicaid, there are other programs to help with out-of-pocket costs for Medicare. My income is $1,600 a month. Would I get any help?

- Topic: Medicare Cost Sharing Assistance

51. Describe in detail the counselor's response when asked, "I was told that if I'm not eligible for Medicaid, there are other programs to help with out-of-pocket costs for Medicare. My income is $1,600 a month. Would I get any help?"

Scoring Guide

1. Accurate and Complete if:
2. Substantive but Incomplete
3. Not Substantive
4. Incorrect
5. Needs Review
    a. Review of Question 51
    b. Reviewer instructions:
        i. Score as accurate and complete if counselor discussed specific Medicare Savings Programs (QMB, SLMB, Extra Help)" by name or by description and said the shopper would probably/may qualify for those. Score as accurate and complete if both an MSP *and* Extra Help was mentioned in name or by description.
        ii. Score as "substantive but not complete" if some but not all accurate information was provided, including if the counselor said that assistance is available without elaborating, mentioned programs other than MSP/Extra Help (e.g., charities, local assistance programs), or if either an MSP OR Extra Help (not both) was mentioned by name or by description.
        iii. Score as "not substantive" if counselor said they do not know, directed the shopper to check with Medicaid, or otherwise did not substantively answer the question.
        iv. Score as incorrect if incorrect information was provided, and the incorrect information was substantive enough to materially change the substance of the counselor's answer or

affect an enrollee’s coverage decision (including if the counselor said the shopper would need to pay the full cost).

v. Score Missing/N/A if shopper could not ask the question.

Medicare-Only Scenario

Question Group 9: Questions 52 – 54

Script Questions/Topics

Medicare-Only Question #2: Do I have to sign up for Medicare when I turn 65, or can I remain on my employer plan?

Topic: Medicare Enrollment & Interaction with Employer Plan

52. Select the option that best describes the counselor’s response when asked, “Do I have to sign up for Medicare when I turn 65, or can I remain on my employer plan?”
    1. No, you don’t have to sign up for Medicare, but you may face a late enrollment penalty if you delay, depending on the size of your employer
    2. No, you don’t have to sign up for Medicare, but you may face a late enrollment penalty if you delay
    3. No, you don’t have to sign up for Medicare
    4. Yes, you have to sign up for Medicare
    5. None of the above
    6. N/A – Dual Eligible scenario or I could not reach a counselor after two attempts

53. Did the counselor mention that if you enroll in Medicare, you can also remain covered by your employer plan?
    1. Yes
    2. No
    3. N/A – Dual Eligible scenario or I could not reach a counselor after two attempts

54. Describe in detail the counselor’s response when asked, “Do I have to sign up for Medicare when I turn 65, or can I remain on my employer plan?”

Scoring Guide

1. Accurate and Complete if:
    a. Question 52: 1 or 2
    b. AND
    c. Question 53: 1
2. Substantive but Incomplete
    a. Question 52: 1 or 2
    b. AND
    c. Question 53: 2
3. Not Substantive
4. Incorrect
5. Needs Review
    a. Question 52: 3 or 4 or 5
    b. Reviewer instructions:
        i. Score as accurate and complete if counselor’s answer substantively makes clear that shopper is not required to enroll in Medicare and can keep their employer plan, and also discusses Part B implications (e.g., mentions possible late enrollment penalty).
        ii. Score as substantive but incomplete if counselor provides some but not all accurate information; for example, if the counselor says that the shopper can remain on employer plan and is not required to enroll in Medicare but does not discuss Part B/late enrollment penalty implications or says that shopper is not required to enroll in Medicare without elaborating.
        iii. Score as “not substantive” if counselor said they do not know or otherwise did not substantively answer the question.

iv. Score as incorrect if incorrect information was provided, and the incorrect information was substantive enough to materially change the substance of the counselor's answer or affect an enrollee's coverage decision (including if the counselor said that the shopper would be automatically enrolled in Medicare, or that shopper is required to enroll in Medicare).
v. Score Missing/N/A if shopper could not ask the question.

Question Group 10: Questions 55, 57, 58, 60

Script Questions/Topics

Medicare-Only Question #3: I keep hearing about Medicare Advantage. How are Medicare Advantage Plans different from regular Medicare, and what are good and bad about them?

Topic: Considerations for Choosing Traditional Medicare vs. Medicare Advantage

55. Which of the following did the counselor mention about Medicare Advantage Plans when asked, "How are Medicare Advantage Plans different from regular Medicare, and what are good and bad about them?"
    1. May have lower premiums, deductibles, and cost sharing than traditional Medicare plus Supplement plan
    2. Has a more restrictive choice of providers and hospitals
    3. You may not be able to keep your doctor
    4. Covers all Medicare Part A and Part B (sometimes Part D) benefits
    5. One must continue to pay the Medicare Part B premium in addition to their Medicare Advantage Plan premium
    6. Often offers additional benefits such as vision, hearing, dental, fitness, grocery, OTC card, etc.
    7. None of the above
    8. N/A – Dual Eligible scenario or I could not reach a counselor after two attempts
56. Not scored
57. What did the counselor say about Medicare Advantage Plans (Part C) when asked, "How are Medicare Advantage Plans different from regular Medicare, and what are good and bad about them?"
58. Which of the following did the counselor mention about original Medicare when asked, "How are Medicare Advantage Plans different from regular Medicare, and what are good and bad about them?"
    1. You automatically enroll in Medicare Part A when you apply for Medicare, which provides coverage for hospital stay
    2. You have the option to enroll in Medicare Part B to cover doctors' services, outpatient care, and preventative services
    3. Part A has a deductible and some cost sharing but no monthly premium
    4. Part B has a monthly premium, some cost sharing, and a small deductible
    5. Premiums and cost sharing can be more expensive than with Medicare Advantage Plans
    6. Your choice of almost any provider, hospital, etc.
    7. In order to receive coverage for prescription drugs, you must obtain a separate Prescription Drug Plan (Part D)
    8. Can be paired with a Medigap/Medicare Supplement policy to reduce premiums, deductibles, and cost sharing
    9. None of the above
    10. N/A – Dual Eligible scenario or I could not reach a counselor after two attempts
59. Not scored
60. What did the counselor say about original Medicare (Part A and Part B) when asked, "How are Medicare Advantage Plans different from regular Medicare, and what are good and bad about them?"
61. Not scored

Scoring Guide

1. Accurate and Complete if:
    a. Question 55: 1 and 2 and 3 and 4 and 5 and 6
    b. And
    c. Question 58: 1 and 2 and 3 and 4 and 5 and 6 and 7 and 8

2. Substantive but Incomplete
    a. Question 55: 1 or 2 or 3 or 4 or 5 or 6
    b. OR
    c. Question 58: 1 or 2 or 3 or 4 or 5 or 6 or 7 or 8
3. Not Substantive
4. Incorrect
5. Needs Review
    a. Needs review of Question 57 and 60 if:
        i. Question 55: 7
        ii. AND
        iii. Question 58: 9
    b. Reviewer instructions:
        i. Score as accurate and complete if all of the information in multiple choice answers 1 – 6 of Question 55 and 1 – 8 of Question 58 are substantively discussed, even if counselor does not use the same wording.
        ii. Score as substantive but incomplete if some (but not all) of the information in multiple choice answers 1 – 6 of Question 55 and 1 – 8 of Question 58 is substantively discussed.
        iii. Score as not substantive if none of the information in multiple choice answers 1 – 6 of Question 55 or 1 – 8 of Question 58 was substantively discussed, if counselor said they do not know, or otherwise did not substantively answer the question.
        iv. Score as incorrect if incorrect information was provided, and the incorrect information was substantive enough to materially change the substance of the counselor's answer or affect an enrollee's coverage decision.
        v. Score Missing/N/A if shopper could not ask the question.

Question Group 11: Questions 62 & 64

Script Questions/Topics

Medicare-Only Question #4: What is a Medicare Supplement Plan, how much do they cost, and when do I enroll?

- Topic: Considerations for Medicare Supplement Plans

62. Which of the following did the counselor mention about Medicare Supplement Plans when asked, "What is a Medicare Supplement Plan, how much do they cost, and when do I enroll?"
    1. Supplement plans can be purchased separately if you choose traditional Medicare to help cover out-of-pocket costs
    2. All supplement plans cover Part A and Part B copays and hospital and hospice costs
    3. Some supplement plans cover Part A and Part B deductibles
    4. Some supplement plans have out-of-pocket limits
    5. Supplement plan costs vary based on how much is covered but usually range from $50-$300 per month
    6. You can enroll in a Supplement plan at any time
    7. You will get better rates if you enroll during your open enrollment period (which begins the day you turn 65 and lasts for six months afterwards)
    8. None of the above
    9. N/A – Dual Eligible scenario or I could not reach a counselor after two attempts
63. Not scored
64. Describe in detail the counselor's response when asked, "What is a Medicare Supplement Plan, how much do they cost, and when do I enroll?"

Scoring Guide

1. Accurate and Complete if:
    a. Question 62: 1 and 2 and 3 and 4 and 5 and 6 and 7
2. Substantive but Incomplete

    a. Question 62: 1 or 2 or 3 or 4 or 5 or 6 or 7
3. Not Substantive
4. Incorrect
5. Needs Review
    a. Needs review of Question 64 if Question 62: 8
    b. Reviewer instructions:
        i. Score as accurate and complete if all of the information in multiple choice answers 1 – 7 of Question 62 was substantively discussed, even if counselor did not use the same wording.
        ii. Score as substantive but incomplete if some (but not all) of the information in multiple choice answers 1 – 7 of Question 62 was substantively discussed.
        iii. Score as not substantive if none of the information in multiple choice answers 1 – 7 of Question 62 was substantively discussed, if counselor said they do not know, or otherwise did not substantively answer the question.
        iv. Score as incorrect if incorrect information was provided, and the incorrect information was substantive enough to materially change the substance of the counselor's answer or affect an enrollee's coverage decision.

<u>Question Group 12: Questions 65 & 66</u>

Script Questions/Topics

Medicare-Only Question #5: Do either regular Medicare, Medicare Advantage Plans, or Medicare Supplement Plans cover long-term care?

- Topic: Coverage of Long-Term Care

65. Select the option that best describes the counselor's response when asked, "Do either original Medicare, Medicare Advantage Plans, or Medicare Supplement Plans cover long-term care?"
    1. In general, none of these cover long-term care, although some time-limited nursing facility stays or in-home services may be covered
    2. No, none of these provide coverage for long-term care
    3. Yes, under one or more of these, long-term care is covered
    4. None of the above
    5. N/A – Dual Eligible scenario or I could not reach a counselor after two attempts
66. Describe in detail the counselor's response when asked, "Do either original Medicare, Medicare Advantage Plans, or Medicare Supplement Plans cover long-term care?"

Scoring Guide

1. Accurate and Complete if:
    a. Question 65: 1 or 2
2. Substantive but Incomplete
3. Not Substantive
4. Incorrect
5. Needs Review
    a. Needs review of Question 66 if Question 65: 3 or 4
    b. Reviewer instructions:
        i. Score as accurate and complete if counselor's answer is substantively the same as multiple choice answer 1 or 2 for Question 65, including if counselor says that Medicare covers nursing facility stays under certain circumstances *and* details the time limitations.
        ii. Score as substantive but not complete if some, but not all relevant information was provided (for example, if the counselor accurately raised the limited Medicare coverage that may be provided in certain situations that is similar to long-term care without clarifying that Medicare does not actually cover long-term care.)

v. Score as not substantive if the counselor said they do not know, or otherwise did not substantively answer the question.
vi. Score as incorrect if incorrect information was provided, and the incorrect information was substantive enough to materially change the substance of the counselor's answer or affect an enrollee's coverage decision, including if the counselor said that Medicare does cover long-term care without elaborating on the limited circumstances that apply.
vii. Score Missing/N/A if shopper could not ask the question.

<u>Question Group 13: Questions 67 & 71</u>

Script Questions/Topics

Medicare-Only Question #6: What are my options for Medicare prescription drug coverage, and how should I choose?

- Topic: Considerations for Prescription Drug Coverage

67. Which of the following considerations did the counselor mention when asked, "What are my options for Medicare prescription drug coverage, and how should I choose?"
    1. You can choose either a Medicare Advantage Plan that covers prescription drugs or a stand-alone Part D plan
    2. Most Medicare Advantage options include prescription drug (Part D) coverage
    3. If you choose original Medicare, you could purchase a separate prescription drug (Part D) plan
    4. Each plan has a different list of covered drugs, and you would choose based on the medicine you take
    5. Each plan has different out-of-pocket costs for different drugs, and you would choose based on the medicine you take and your comfort with costs
    6. None of the above
    7. N/A – Dual Eligible scenario or I could not reach a counselor after two attempts
68. Not scored
69. Not scored
70. Not scored
71. Describe in detail the counselor's response when asked, "What are my options for Medicare prescription drug coverage, and how should I choose?"

Scoring Guide

1. Accurate and Complete if:
    a. Question 67: 1 and 2 and 3 and 4 and 5
2. Substantive but Incomplete
    a. Question 67: 1 or 2 or 3 or 4 or 5
3. Not Substantive
4. Incorrect
5. Needs Review
    a. Needs review of Question 71 if Question 67: 6
    b. Reviewer instructions:
        i. Score as accurate and complete if all of the information in multiple choice answers 1 – 5 of Question 67 was substantively discussed, even if the counselor did not use the same wording.
        ii. Score as substantive but incomplete if some (but not all) of the information in multiple choice answers 1 – 5 of Question 67 was substantively discussed
        iii. Score as not substantive if none of the information in multiple choice answers 1 – 5 of Question 67 was substantively discussed, if counselor said they do not know, or otherwise did not substantively answer the question.

iv. Score as incorrect if incorrect information was provided, and the incorrect information was substantive enough to materially change the substance of the counselor’s answer or affect an enrollee’s coverage decision.
v. Score Missing/N/A if shopper could not ask the question.

Question Group 14: Question 72

Script Questions/Topics

Medicare-Only Question #7: My friend told me about [plan name listed in your shop confirmation]. Can you tell me if [name of doctor listed in your shop confirmation] is in the network there?

- Topic: Ability to Determine if Specific PCP is in Network for Specific Plan

72. What was the counselor’s response when asked, “My friend told me about [plan name]. Can you tell me if [doctor name] is in the network there?”
    1. The doctor is in the network
    2. The doctor is not in the network
    3. Neither of the above
    4. N/A – Dual Eligible scenario or I could not reach a counselor after two attempts
73. Not scored
74. Not scored

Scoring Guide

1. Accurate and Complete if:
    a. Question 72: 1
2. Substantive but Incomplete
3. Not Substantive
    a. Question 72: 3
4. Incorrect
    a. Question 72: 2
5. Needs Review
    a. None

Question Group 15: Question 75

Script Questions/Topics

Medicare-Only Question #8: What is the monthly premium for [plan name listed in your shop confirmation]?

- Topic: Ability to Determine Premium for Specific Plan

75. What dollar amount(s) did the counselor provide for the monthly premium, or if the monthly premium was not provided, what did the counselor say when asked, “What is the monthly premium for [plan name]?

Scoring Guide

1. Accurate and Complete if:
2. Substantive but Incomplete
3. Not Substantive
4. Incorrect
5. Needs Review
    a. Needs review of Question 75
    b. Reviewer instructions:

i. Score as accurate and complete if counselor provided the Plan premium and the Part B premium, and the amounts for each were accurate for the year in which the shop took place.
ii. Score as substantive but incomplete if the counselor only provided *either* the plan premium *or* the Part B premium (without clarifying that the plan premium was $0) and the amount provided was accurate for the year in which the shop took place; or provided an accurate range.
vi. Score as not substantive if the counselor did not provide an amount or range, said they do not know, or otherwise did not substantively answer the question.
iii. Score as incorrect if the counselor provided a dollar amount that was not accurate for the year in which the shop took place, or otherwise provided incorrect information which was substantive enough to materially change the substance of the counselor's answer or affect an enrollee's coverage decision.
iv. Score Missing/N/A if shopper could not ask the question.

<u>Question Group 16: Question 76 & 77</u>

Script Questions/Topics

Medicare-Only Question #9: Would [plan name listed in your shop confirmation] let me go out of network?

- Topic: Ability to Determine if Specific Plan Allows Out-of-Network Care

76. What was the counselor's response when asked, "Would [plan name] let me go out of network?"
    1. PPO: Yes, but with higher cost sharing
    2. PPO: Yes, with no mention of higher cost sharing
    3. PPO: No, plan would not allow me to go out of network
    4. HMO: No, plan would not allow me to go out of network
    5. HMO: Yes, but with higher cost sharing
    6. HMO: Yes, with no mention of higher cost sharing
    7. None of the above
    8. N/A – Dual Eligible scenario or I could not reach a counselor after two attempts
77. Describe in detail the counselor's response when asked, "Would [plan name] let me go out of network?"
78. Not scored

Scoring Guide

1. Accurate and Complete if
    a. Question 76: 1 or 4
2. Substantive but Incomplete
    a. Question 76: 2
3. Not Substantive
4. Incorrect
    a. Question 76: 3
5. Needs Review
    a. Needs review of Question 77 if Question 76: 5 or 6 or 7
    b. Reviewer instructions:
        i. Score as accurate and complete if the plan being asked about is a PPO plan, and the counselor said yes and specified that there would be a higher cost or copay; or if the plan being asked about is an HMO and the counselor said no.
        ii. Score as substantive but incomplete if the plan being asked about is a PPO plan, and the counselor said yes but did not specify that the copay or cost would be higher; or if counselor did not say yes or no but said the copay or cost would be higher.
        vii. Score as not substantive if the counselor said they do not know, or otherwise did not substantively answer the question.
        iii. Score as incorrect if incorrect information was provided, and the incorrect information was substantive enough to materially change the substance of the

counselor's answer or affect an enrollee's coverage decision, including if the counselor said that the plan being asked about is not available in the coverage area.

<u>Question Group 17: Question 79</u>

Script Questions/Topics

Medicare-only question #10: What is the copay for a primary care doctor visit for [plan name listed in your shop confirmation]?

- Topic: Knowledge of in-network PCP copay

79. What was the copay amount given for an in-network primary care doctor visit, or what was said about in-network primary care doctor visits if no amount was given?

80. Not scored
81. Not scored
82. Not scored
83. Not scored
84. Not scored
85. Not scored

Scoring Guide

1. Accurate and Complete if
2. Substantive but Incomplete
3. Not Substantive
4. Incorrect
5. Needs Review
    a. Needs review of Question 79
    b. Reviewer instructions:
        i. Score as accurate and complete if the counselor said that the copay for an in-network PCP is $0 or that there is no copay.
        ii. Score as substantive but incomplete if the counselor made a general statement such as "there is no copay for an HMO" or "there is usually a $0 copay" without referencing the specific plan that was asked about, or provided a dollar amount range that includes $0.
        iii. Score as not substantive if the counselor said that they do not know, or otherwise did not answer the question.
        iv. Score as incorrect if incorrect information was provided, and the incorrect information was substantive enough to materially change the substance of the counselor's answer or affect an enrollee's coverage decision, including if the counselor said a dollar amount other than $0 or provided a range that did not include $0.
        v. Score Missing/N/A if shopper could not ask the question.

<u>Question Group 18: Question 86 & 87</u>

Script Questions/Topics

Medicare-Only Question #12: For [plan name listed in your shop confirmation], is there a limit on out-of-pocket costs I would have to pay each year?

- Topic: Knowledge of maximum out-of-pocket limit

86. Which of the following best describes the counselor's response when asked, "For [plan name], is there a limit on out-of-pocket costs I would have to pay each year?"

1. PPO: The counselor provided two separate dollar amounts: one for in-network care and one for out-of-network care and specified that certain costs do not count toward the limits, including amounts you pay in premiums and for prescription drugs
2. PPO: The counselor provided two separate dollar amounts: one for in-network care and one for out-of-network care, but did not specify that certain costs do not count toward the limits
3. PPO: The counselor provided a dollar amount but did not differentiate between in-network or out-of-network
4. PPO: The counselor indicated there is a maximum, but did not provide a dollar amount
5. PPO: The counselor said there is no maximum
6. HMO: The counselor provided a dollar amount and specified that certain costs do not count toward the limit, including amounts you pay in premiums, for prescription drugs, or for out-of-network care
7. HMO: The counselor provided a dollar amount, but did not specify which costs do not count toward the limit
8. HMO: The counselor indicated there is a maximum, but did not provide a dollar amount
9. HMO: The counselor said there is no maximum
10. None of the above
11. Provided the wrong dollar amount
12. N/A – Dual Eligible scenario or I could not reach a counselor after two attempts

87. What dollar amount(s) did the counselor provide for the maximum out-of-pocket costs, or if no dollar amount was given, what did the counselor say when asked, "For [plan name], is there a limit on out-of-pocket costs I would have to pay each year?"

Scoring Guide

1. Accurate and Complete if
    a. Question 86: 1 or 2 or 3 or 6 or 7
2. Substantive but Incomplete
    a. Question 86: 4 or 8
3. Not Substantive
    a. Question 86: 10
4. Incorrect
    a. Question 86: 5
5. Needs Review
    a. Needs review of Question 87 (and 2023/2024 Medicare.gov plan finder information) if Question 86: 11.
    b. Reviewer instructions:
        i. Score as accurate and complete if the dollar amount provided by the SHIP counselor matches the amount included in Medicare.gov plan finder tool for the year in which the shop took place.
        ii. Score as incorrect if the dollar amount provided by the SHIP counselor does not match the amount included in Medicare.gov plan finder tool for the year in which the shop took place.
        iii. Score Missing/N/A if shopper could not ask the question.

Question Group 19: Question 88 & 89

Script Questions/Topics

Medicare-Only Question #13: Does [plan name listed in your shop confirmation] include prescription drug coverage?

- Topic: Ability to determine if specific plan includes coverage for prescription drugs

88. What was the counselor's response when asked, "Does [plan name] include prescription drug coverage?"
    1. The plan includes prescription drug coverage
    2. The plan does not include prescription drug coverage
    3. Neither of the above

4. N/A – Dual Eligible scenario or I could not reach a counselor after two attempts

89. Describe in detail the counselor's response when asked, "Does [plan name] include prescription drug coverage?"

Scoring Guide

1. Accurate and Complete if
   a. Question 88: 1
2. Substantive but Incomplete
3. Not Substantive
4. Incorrect
   a. Question 88: 2
5. Needs Review
   a. Needs review of Question 89 if Question 88: 3
   b. Reviewer instructions
      i. Score as accurate and complete if counselor says yes
      ii. Score as substantive but incomplete if counselor provides a correct answer that is not specific to the plan being asked about (e.g., says they believe so or that most plans do) or says that the specific drugs are covered without clarifying that the plan includes prescription drug coverage more generally.
      iii. Score as not substantive if the counselor said that they do not know, or otherwise did not answer the question.
      iv. Score as inaccurate if the counselor said no, or otherwise provided incorrect information that was substantive enough to materially change the substance of the counselor's answer or affect an enrollee's coverage decision.
      v. Score Missing/N/A if shopper could not ask the question.

<u>**Question Group 20: Question 90 – 92**</u>

Script Questions/Topics

Medicare-Only Question #14a: I take Lipitor. Is that covered by [plan name listed in your shop confirmation]?

- **Follow-up question #14b:** If the generic version is not mentioned, you must also ask, "Is a generic version of Lipitor covered? I would be willing to take that."
- Topic: Ability to determine if plan covers specific drug (Lipitor) and/or its generic equivalent.

90. Select the option that best describes the counselor's response when asked, "I take Lipitor. Is that covered by [plan name]?"
    1. No, Lipitor is not covered, but the generic version is covered
    2. No, Lipitor is not covered, and there was no mention of the generic version
    3. Yes, Lipitor is covered
    4. None of the above
    5. N/A – Dual Eligible scenario or I could not reach a counselor after two attempts

91. If the generic version of Lipitor was not mentioned, what did the counselor say when asked if a generic version of Lipitor is covered?
    1. Yes, a generic version is covered
    2. No, a generic version is not covered
    3. Neither of the above
    4. N/A – a generic version was mentioned
    5. N/A – Dual Eligible scenario or I could not reach a counselor after two attempts
92. Describe in detail the counselor's response when asked, "I take Lipitor. Is that covered by [plan name]?"

Scoring Guide

1. Accurate and Complete if
    a. Question 90: 1
    b. Or
    c. Question 90: 2
        i. AND
        ii. Question 91: 1
2. Substantive but Incomplete
3. Not Substantive
4. Incorrect
5. Needs Review
    a. Needs review of Question 91 and 92 if
        i. Question 90: 3 or 4
        ii. OR
        iii. Question 90: 2
            1. AND
            2. Question 91: 2 or 3 or 4
    b. Reviewer instructions
        i. Score as accurate and complete if counselor substantively said that Lipitor is not covered but a generic version is, or that Lipitor is covered but a generic version is available at a lower cost.
        ii. Score as substantive but not complete if the counselor said that Lipitor is covered, without elaborating on cost or clarifying whether they were referring to Liptior itself, or its generic.
        iii. Score as not substantive if the counselor said they did not know, said something to the effect of that they thought so (without looking up the answer) or otherwise did not answer the question.
        iv. Score as inaccurate if the counselor provided incorrect information that was substantive enough to materially change the substance of the counselor's answer or affect an enrollee's coverage decision, including if the counselor said that neither Lipitor nor its generic was covered.
        v. Score Missing/N/A if shopper could not ask the question.
