# Supplementary material for: Accuracy of Medicare Information Provided by State Health Insurance Assistance Programs
Source: JAMA Netw Open. 2025 Apr 1;8(4):e252834. doi: 10.1001/jamanetworkopen.2025.2834 (PMC11962663; doi:10.1001/jamanetworkopen.2025.2834)
Supplement: Supplement 2. — Data Sharing Statement [file jamanetwopen-e252834-s002.pdf]

Data Sharing Statement

Dugan. Accuracy of Medicare Information Provided by State Health Insurance Assistance Programs. *JAMA Netw Open*. Published April 01, 2025.
doi:10.1001/jamanetworkopen.2025.2834

Data
Data available: Yes
Data types: Deidentified participant data
How to access data: Please email garrido@bu.edu for data
When available: With publication

Supporting Documents
Document types: None

Additional Information
Who can access the data: Researchers requesting the data
Types of analyses: Any purpose
Mechanisms of data availability: After approval of a proposal
